# Alkali and alkaline-earth metal hybrid doping enhances NLO response: from a weak alkaline-earthide of (Be^*δ*−^@C_50_N_5_H_5_)^(*δ*′−*δ*)−^‒M^*δ*′+^ to dual-alkali-metal salts of (Mg^*δ*+^@C_50_N_5_H_5_)^(*δ*+*δ′*)^−^^–M^*δ'*+^ and (Ca^2+^@C_50_N_5_H_5_)^(2+*δ*)−^–M^*δ*+^

**DOI:** 10.1039/d6ra04746g

**Published:** 2026-08-18

**Authors:** Feng Xu, Zhi-Xuan Wang, Rui-Wen Liu, Hao Chen, Jin-Jin Wei, Zhijun Wang, Wen Zhang, Hong-Liang Xu, Yin-Feng Wang

**Affiliations:** a Key Laboratory of Jiangxi Province for Special Optoelectronic Artificial Crystal Materials, Key Laboratory of Energy Conversion Optoelectronic Functional Materials of Jiangxi Education Institutes, School of Chemistry and Chemical Engineering. Jinggangshan University Ji'an Jiangxi 343009 P.R. China cyclont@yeah.net; b College of Chemistry, Huazhong Agricultural University Wuhan 430070 P. R China; c Institute of Functional Material Chemistry, National & Local United Engineering Laboratory for Power Batteries, Department of Chemistry, Northeast Normal University Changchun 130024 P. R. China

## Abstract

Aiming to obtain enhanced NLO performance for C_50_N_5_H_5_ cages through hybrid alkali/alkaline-earth metal doping in comparison with dual alkali-metal doping schemes, (M′@C_50_N_5_H_5_)–M (M = Li, Na and K; M′ = Be, Mg and Ca) were theoretically constructed by placing an alkali and alkaline earth metal atom outside and inside the C_50_N_5_H_5_ cage, respectively. Binding characteristic analysis indicates that it changes from a weak alkaline-earthide of (Be^*δ*−^@C_50_N_5_H_5_)^(*δ*′-*δ*)−^–M^*δ*+^ to dual-alkali-metal salts of (Mg^*δ*+^@C_50_N_5_H_5_)^(*δ*+*δ′*)−^–M^*δ′*+^ and (Ca^2+^@C_50_N_5_H_5_)^(2+*δ*)−^–M^*δ*+^ (M = Li, Na and K) as the atomic number of the alkali and alkaline earth metal increases. Notably, our species possess better stability and NLO performance than their alkali analogues, (M@C_50_N_5_H_5_)M′ (M, M′ = Li, Na and K, *J. Mol. Liq.*, 2023, **385**, 122400). The static first hyperpolarizability exhibits an initial decrease followed by a subsequent increase in the (Be@C_50_N_5_H_5_)–M and (Ca@C_50_N_5_H_5_)–M series but increases monotonically in the (Mg@C_50_N_5_H_5_)–M (M = Li, Na and K) series. The alkalide of (Ca^2+^@C_50_N_5_H_5_^(2+*δ*)+^)–K^*δ*+^ holds prominently high *β*_0_ up to 4.05 × 10^5^ a.u. A similar trend has emerged for the dynamic first hyperpolarizability.

## Abstract 2

Hybrid alkali/alkaline-earth metal doping is shown to be a better strategy in comparison with dual alkali-metal doping schemes.

## Graphical abstract

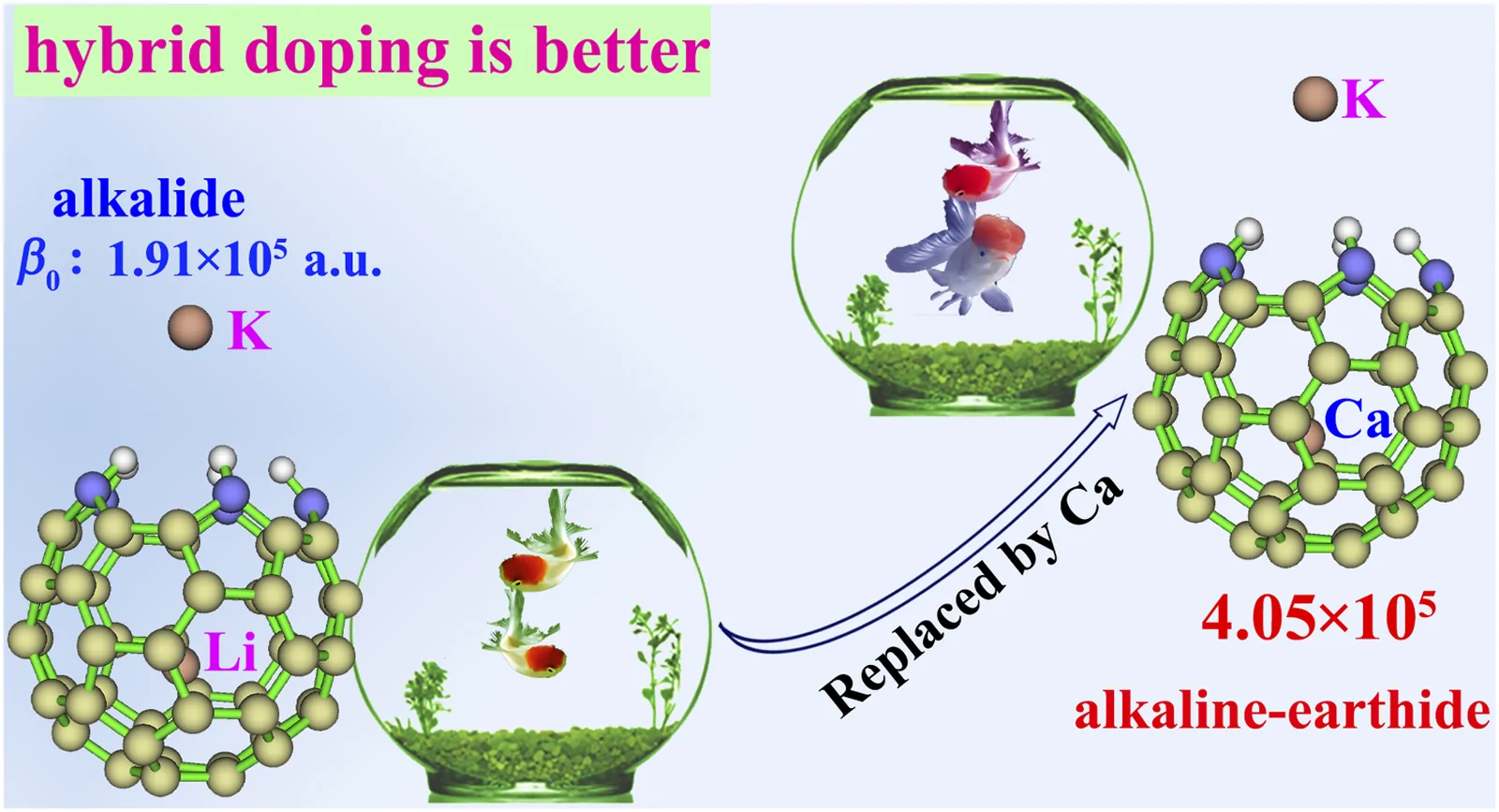

## Introduction

High-performance nonlinear optical (NLO) materials have long been a research hotspot owing to their applications in optical communication, terahertz generation, photonic computing and photonic crystals.^1,2^ Substantial efforts have focused on modulating the static first hyperpolarizability (*β*_0_). For instance, alkali metal fluoroxiborates (AB_4_O_4_X; X = K, Rb, and Cs) have been reported to display prominent second-harmonic generation (SHG);^3^ Ghosal *et al.* further demonstrated that the electronic-structure modulation of tetragonal silicene and germanene quantum dots can boost NLO responses.^4^ Doping with (super)alkali or alkaline-earth metal atoms offers an effective strategy for NLO modulation, attracting extensive research attention.^5–27^ Incorporating s-electron-rich metal atoms into complexants can lead to the emergence of two distinct effects: the salt effect and the alkalide effect. For the former, Xu *et al.* confirmed that lithiation-induced salt effects can enhance *β*_0_ values of acenes and heteronanotubes,^5,6^ and our previous work also revealed considerable *β*_0_ on Li@AR (AR = benzene and naphthalene)^7^ and Ca@SP-Helix (SP-Helix = aza-single-strand pyridine helix).^8^ Alkalides are a unique class of species where alkali metals serve as the anion. Experimental and computational evidence indicates typical alkalides feature superalkali–alkalide interactions,^28^ and they act as potent, regioselective reducing agents.^29^ Numerous alkalide and superalkalide systems have been proposed as NLO candidates,^9–19^ including crown-ether-based alkalides M@[15-crown-5]^12^ and Li_3_O@[12-crown-4]M.^13^ We have reported a series of alkalide-based NLO switches of Li(HF)_3_Li and Li(HCN)_3_Li.^9,10^

Nevertheless, conventional alkali-metal-based alkalides suffer from high chemical reactivity, imposing strict synthetic and storage constraints under ambient air and temperature. To address this issue, alkaline-earthides (alkaline-earth-based analogues) with rigid complexants have been developed.^20–27^ However, published alkaline-earth salt and alkaline-earthide systems generally deliver lower *β*_0_ than their alkali analogues, which is rarely discussed in previous studies. For example, M^+^(calix[4]pyrrole)M′^−^ (3.80 × 10^3^–1.98 × 10^4^ au)^20^ has a narrower *β*_0_ range than Li^+^(calix[4]pyrrole)M^−^ (M = Li, Na and K; M′ = Be, Mg and Ca) (1.10 × 10^4^–3.59 × 10^4^ au).^14^ This raises two open questions: whether hybrid doping outperforms single-type metal doping in NLO performance and whether stable alkaline-earthide/salt can achieve superior NLO responses to alkali analogues.

Motivated by the above issues, we designed a series of (M′@C_50_N_5_H_5_)–M (M = Li, Na and K; M′ = Be, Mg and Ca). The C_50_N_5_H_5_ cage is derived from the synthetically available C_50_H_10_ cage.^30^ Distinct from the reported all-alkali (^+^M@C_50_N_5_H_5_)M′^−^ (M, M′ = Li, Na and K),^19^ our systems feature an alkaline-earth metal encapsulated inside the cage and an alkali metal anchored to the exterior. The calculated results show that these hybrid clusters have improved stability compared with all-alkali analogues and comparable stability to most reported alkalides, as well as appreciable NLO responses. This work provides a theoretical strategy for designing stable, high-efficiency NLO materials and guidance for related experimental syntheses.

### Computational details

The geometric structures and relevant property calculations for (M@C_50_N_5_H_5_)M′ (M, M′ = Li, Na and K)^19^ were obtained with the M06-2X^31^ functional and the 6-31G(d) basis set. To compare, the geometric structures of (M′@C_50_N_5_H_5_)–M (M = Li, Na and K; M′ = Be, Mg and Ca) with all real frequencies were also realized at the M06-2X/6-31G(d) level. The natural population analysis (NPA),^32^ vertical ionization energies (VIE), and interaction energies (*E*_int_) values were obtained at the M06-2X/6-311++G(d,p) level. The VIE was estimated as:

1 VIE = *E*[(M′@C_50_N_5_H_5_)–M]^+^ − *E*[(M′@C_50_N_5_H_5_)–M]

The thermodynamic stabilities of (M@C_50_N_5_H_5_)–M′ are in terms of *E*_int_, which is calculated as:

2 *E*_int_ = *E*_(M′@C_50_N_5_H_5_)–M_ − *E*_M_ − *E*_M′_ − *E*_C_50_N_5_H_5__ + BSSE

where *E*_int_, *E*_(M@C_50_N_5_H_5_)M′_, *E*_M_, *E*_M′_ and *E*_C_50_N_5_H_5__ are, respectively, the interaction energy and the energies of (M@C_50_N_5_H_5_)–M′, the bare alkali (M), the alkaline earth metal atom (M′), and the complexant C_50_N_5_H_5_. The counterpoise-corrected method was used to remove the basis set superposition error (BSSE).^33^ Electron excitations were investigated by time-dependent DFT (TD-DFT) with the 6-311++G(d,p) method. To investigate which excitation path primarily contributes to the total *β*^e^_0_, 250 excited states were considered.

In previous alkalide and related systems, the M06-2X method has been widely used to calculate *β*^e^_0_. Recently, among CAM-B3LYP, LC-BLYP, M06-2X, MP2, MP3, MP4(SDQ), and QCISD methods, the M06-2X results are close to those obtained by the more sophisticated QCISD methods for the alkalide of M(HF)_3_M (M = Na or Li).^9^ Table S1 shows that, at the M06-2X/6-311++G(d,p) level, the static *β*^e^_0_ values of (Be@C_50_N_5_H_5_)–Na and (Be@C_50_N_5_H_5_)–K are 3.74 × 10^3^ and 2.75 × 10^5^ a.u., respectively. Despite similar geometries, the *β*^e^_0_ values vary by over two orders of magnitude, suggesting potential drawbacks in the chosen computational approach. For the alkali and alkaline earth metal doped open-cage fullerenes, this may involve strong static electron-correlation effects. It is known that the static *β* components are obtained from the second derivative of the dipole moment with respect to an electric field, and errors in the dipole moment are nonlinearly amplified. Moreover, functionals with high HF exchange are unsuitable for multireference systems.^34^ So, it is necessary to identify whether the multi-reference characteristics are significant before calculating *β*_0_ values. The fractional orbital density (FOD) analysis^34^ was done at the TPSS/def2-TZVP (*T* = 5000 K) level with a convergence threshold of “tightSCF” and a truncation threshold of “normalPNO” by the ORCA program.^35,36^ The FOD analysis plot at *σ* = 0.003 e Bohr^−3^ (TPSS/def2-TZVP (*T* = 5000 K) level) for (M′@C_50_N_5_H_5_)–M (M = Li, Na and K; M′ = Be, Mg and Ca) is shown in Fig. S1. From Fig. S1, the FOD values are in the range of 2.162 to 3.396. So, it may be a significant yet relatively localized or rather delocalized FOD visible for these (M′@C_50_N_5_H_5_)–M from M/M′ = Li/Be to K/Ca. Then, the frequency-dependent NLO properties of (M′@C_50_N_5_H_5_)–M (M = Li, Na and K; M′ = Be, Mg and Ca) were obtained with the TPSSh method with 10% HF exchange–correlation.

The frequency-dependent *β*(*ω*) can be obtained from:

3 *β*(*ω*) = (*β*_*x*_^2^ + *β*_*y*_^2^ + *β*_*z*_^2^)^1/2^

where *β*_*i*_(*ω*) = [*β*_*ijj*_(−2*ω*;*ω*,*ω*) + 2*β*_*jji*_(−2*ω*;*ω*,*ω*)]/5 (*i*, *j* = *x*, *y*, and *z*) for the second harmonic generation (SHG).^37^

Considering two measurable properties, the projection of *β* onto the dipole moment vector (*β*_prj_) and the hyper Rayleigh scattering^38^ response (*β*_HRS_),^39^*β*(*ω*) was also obtained at the TPSSh/def2-SVP level.

To further investigate the thermodynamic stability of our species, taking (Ca@C_50_N_5_H_5_)–M (M = Li, Na, and K) as examples, we performed AIMD simulations at the B97-3c level of theory. For each molecule under consideration, AIMD simulations were carried out for 4000 fs with a time step of 1 fs and a fixed temperature of 298.15 K and 1 atm pressure with the Canonical Sampling through Velocity Rescaling (CSVR) thermostat^40^ in the ORCA program. This method includes corrections for diverse interactions, which makes it suitable for studying a series of molecular systems with good accuracy.^41^ This trajectory analysis has been suggested to study the dynamic stability of parallel stacked complexes M[9C]2M (M = Li, Na, K, Be, Mg, and Ca).^42^

*β*_HRS_ and *β*_prj_ were obtained from:4
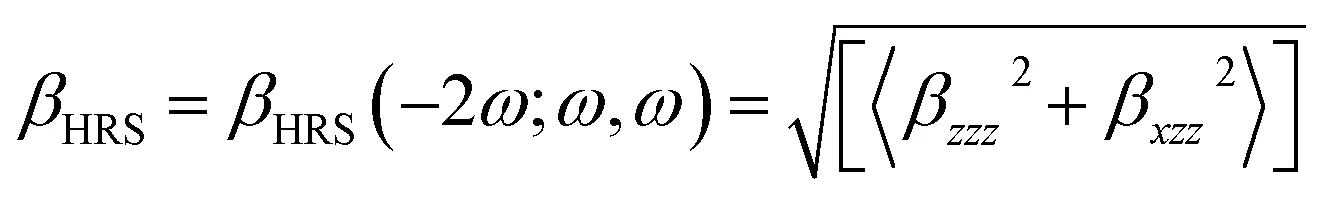
5
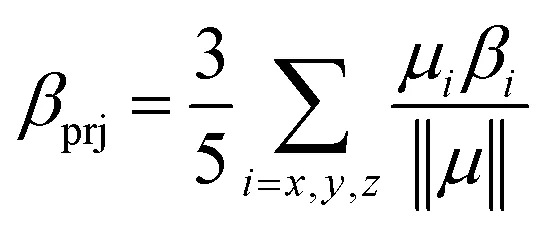


For *β*_0_, *ω* = 0 au. Using the Born–Oppenheimer approximation, *β*_0_ can be mainly written as an electronic contribution (*β*^e^_*ijk*_) and a nuclear relaxation contribution (*β*^nr^_*ijk*_, *i*, *j*, *k* = *x*, *y*, *z*). *β*^nr^_*ijk*_ is given in terms of vibronic energies and dipole moment matrix elements over the vibronic wave functions.^43,44^ Details of *β*^nr^_*ijk*_ and *β*^e^_*ijk*_ can be found in the SI.

All quantum chemistry calculations were performed using the Gaussian 16 package.^45^ After calculations, the data analysis, including maps of electron density difference (EDD),^46^ charge-transfer spectra (CTS),^47^ and the hyperpolarizability values, were obtained using the Multiwfn 3.8(dev) code.^48,49^

## Results and discussion

### Optimized geometries and stabilities

**Fig. 1:**
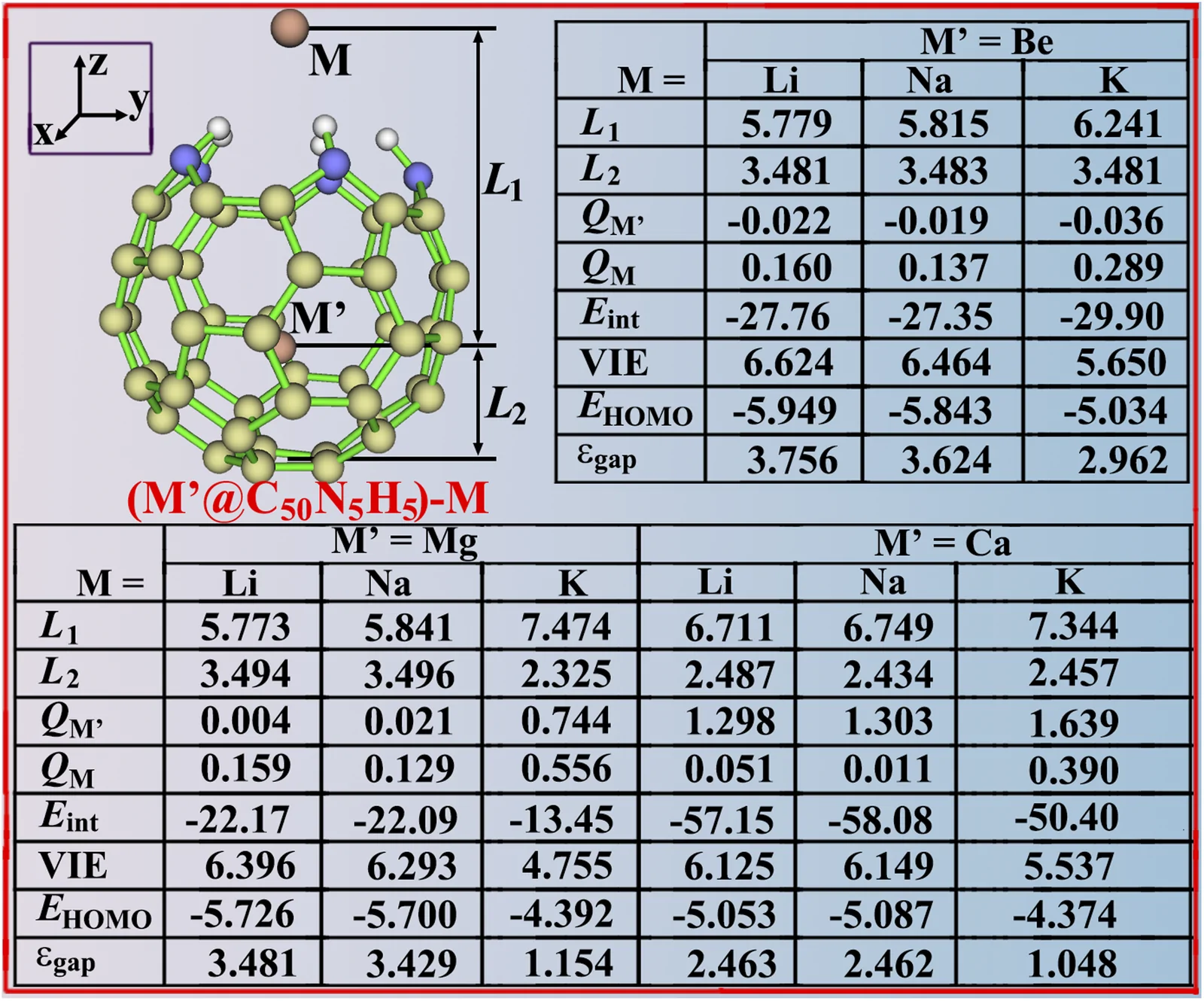
Optimized geometries of (M′@C_50_N_5_H_5_)–M (M = Li, Na and K; M′ = Be, Mg and Ca). Selected bond lengths (*L*_1_ and *L*_2_, Å), NPA charges of M and M′ (*Q*_M_ and *Q*_M′_, |*e*|), interaction energies (*E*_int_, kcal mol^−1^), vertical ionization energies (VIE, eV), and HOMO–LUMO gaps (*ε*_gap_, eV).

After trapping an alkaline earth atom into the C_50_N_5_H_5_ cage and doping an alkali earth atom over the N_5_H_5_ subunit, structures of (M′@C_50_N_5_H_5_)–M (M = Li, Na and K; M′ = Be, Mg and Ca) with all real frequencies were obtained. From Fig. 1, the M–M′ distance (*L*_1_) increases as the atomic number of M increases in each series (Be@C_50_N_5_H_5_)–M, (Mg@C_50_N_5_H_5_)–M and (Ca@C_50_N_5_H_5_)–M (M = Li, Na and K). *L*_2_ represents the height at which the alkaline earth metal atoms leave the bottom of the cage. Note that *L*_2_ is higher for (Be@C_50_N_5_H_5_)–M, (Mg@C_50_N_5_H_5_)–Li and (Mg@C_50_N_5_H_5_)–Na than for (Mg@C_50_N_5_H_5_)–K and (Ca@C_50_N_5_H_5_)–M (M = Li, Na and K). *L*_2_ values are, respectively, in the ranges of 3.481–3.496 Å and 2.325–2.487 Å for the former and the latter. Unfortunately, we do not find any isomer with a different *L*_2_ value. So, the difference in *L*_2_ values arises from the balance of different forces.

As an NLO molecular material, we are also concerned about its chemical and electronic stability, which reflect the tendency of a molecule to transform into another molecule or to lose an electron, respectively. Molecular electronic, chemical, and thermodynamic stability can be characterized by VIE, *ε*_gap_, and *E*_int_, respectively. For the open-shell systems of (Ca@C_50_N_5_H_5_)–M (M = Li, Na and K), *ε*_gap_ gives the difference in energies between the HOMO(α) and LUMO(α). The data in Fig. 1 show that, for each series of (Be@C_50_N_5_H_5_)–M, (Mg@C_50_N_5_H_5_)–M, and (Ca@C_50_N_5_H_5_)–M (M = Li, Na and K), an almost monotonic decrease is observed for both VIE and *ε*_gap_ values as the atomic number of the alkali metal atom (M) increases. So, both electronic and chemical stability monotonically decrease for these series as the atomic number of M increases. The VIE and *ε*_gap_ values are, respectively, in the ranges of 5.650–6.624 eV and 2.962–3.756 eV for (Be@C_50_N_5_H_5_)–M, (Mg@C_50_N_5_H_5_)–Li, and (Mg@C_50_N_5_H_5_)–Na with higher *L*_2_, while they are in the ranges of 4.755–6.149 eV and 1.154–2.463 eV for (Mg@C_50_N_5_H_5_)–K and (Ca@C_50_N_5_H_5_)–M (M = Li, Na and K) with lower *L*_2_, respectively. Thus, the higher the height of the alkaline-earth metal from the bottom, the higher the electronic and chemical stability of the structure.

As illustrated in Fig. 1, two distinct structural categories can be classified according to the variation in the *L*_2_ geometric parameter. The first category features large *L*_2_ values ranging from 3.481 to 3.496 Å, covering all (Be@C_50_N_5_H_5_)–M, (Be@C_50_N_5_H_5_)–M, (Mg@C_50_N_5_H_5_)–Li, and (Mg@C_50_N_5_H_5_)–Na. The second category exhibits obviously reduced *L*_2_ values of 2.325–2.487 Å, including (Mg@C_50_N_5_H_5_)–K and all (Ca@C_50_N_5_H_5_)–M (M = Li, Na and K). Excluding the special case of (Mg@C_50_N_5_H_5_)–K, a clear structure–property correlation is observed: species with larger *L*_2_ distances exhibit smaller interaction energies (|*E*_int_| of 22.09–29.90 kcal mol^−1^) and wider band gaps (*ε*_gap_ of 2.962–3.756 eV), whereas those with shorter *L*_2_ distances possess stronger binding (|*E*_int_| of 50.40–58.08 kcal mol^−1^) and narrower band gaps (*ε*_gap_ of 1.048–2.463 eV). Accordingly, the former species with larger *L*_2_ distances show inferior thermodynamic stability but improved chemical stability, while the latter species with smaller *L*_2_ values feature superior thermodynamic stability accompanied by reduced chemical stability.

Among all nine studied species, (Mg@C_50_N_5_H_5_)–K presents the minimum *L*_2_ distance (2.325 Å) and the maximum *L*_1_ distance (7.474 Å). Consequently, it possesses the narrowest *ε*_gap_ value (1.154 eV) and the smallest |*E*_int_| value (13.45 kcal mol^−1^), demonstrating that (Mg@C_50_N_5_H_5_)–K features the poorest thermodynamic and chemical stability among the nine species of (M′@C_50_N_5_H_5_)–M (M = Li, Na and K; M′ = Be, Mg and Ca). The comparison between (Mg@C_50_N_5_H_5_)–Na (longer *L*_2_) and (Mg@C_50_N_5_H_5_)–K (shorter *L*_2_) shows that the latter displays a narrower *ε*_gap_ value, which is consistent with the above-mentioned correlation trend. However, its |*E*_int_| behavior deviates from the above-predicted rule: despite its shorter *L*_2_ distance, which favors stronger binding, (Mg@C_50_N_5_H_5_)–K delivers a weaker *E*_int_, suggesting the presence of unique underlying mechanisms.

For all (Ca@C_50_N_5_H_5_)–M (M = Li, Na and K), the encadpsulated Ca atom carries highly positive NPA charges exceeding 1.0|*e*|. In contrast, the Mg atom in (Mg@C_50_N_5_H_5_)–K only presents a moderate positive NPA charge of 0.744|*e*| (see Fig. 1). This significant charge difference demonstrates that Ca nearly donates both 4s valence electrons to the C_50_N_5_H_5_ cage, corresponding to an effective oxidation state of +2, while the Mg atom only transfers one 3s valence electron, yielding a lower oxidation state of +1. Given that electrostatic interaction strength is proportional to the square of the transferred charge, the Ca-cage electrostatic interaction in (Ca@C_50_N_5_H_5_)–M (M = Li, Na and K) is far stronger than the Mg-cage electrostatic interaction in (Mg@C_50_N_5_H_5_)–K. Notably, although the short *L*_2_ distance (2.325 Å) of (Mg@C_50_N_5_H_5_)–K is geometrically favourable for electrostatic stabilization compared with the longer *L*_2_ distances of all (Ca@C_50_N_5_H_5_)–M (2.434–2.487 Å, M = Li, Na and K), this structural advantage may be largely offset. The shorter *L*_2_ distance in (Mg@C_50_N_5_H_5_)–K can bring a stronger repulsion between the Mg atom and the bottom of the cage than that between the Ca atom and the bottom of the cage in (Ca@C_50_N_5_H_5_)–M, which obviously undermines structural stability. Collectively, the weaker electrostatic stabilization and the additional destabilizing repulsive component result in a much smaller |*E*_int_| value (13.45 kcal mol^−1^*vs.* 50.40–58.08 kcal mol^−1^) and inferior thermodynamic stability of (Mg@C_50_N_5_H_5_)–K relative to all (Ca@C_50_N_5_H_5_)–M (M = Li, Na and K).

In comparison with all (Ca@C_50_N_5_H_5_)–M (M = Li, Na and K), the species of (Be@C_50_N_5_H_5_)–M (M = Li, Na and K), (Mg@C_50_N_5_H_5_)–Li and (Mg@C_50_N_5_H_5_)–Na exhibit evidently longer *L*_2_ distance (3.481–3.496 Å *vs.* 2.434–2.487 Å) and negligible alkaline-earth metal NPA charges (−0.019–0.021|*e*| *vs.* 1.298–1.639|*e*|). These two factors jointly weaken the electrostatic interactions in these Be- and Mg-based systems, leading to lower |*E*_int_| values relative to their Ca-based counterparts (22.09–29.90 kcal mol^−1^*vs.* 50.40–58.08 kcal mol^−1^). As a result, (Ca@C_50_N_5_H_5_)–M possesses distinctly higher thermodynamic stability than (Be@C_50_N_5_H_5_)–M (M = Li, Na and K), (Mg@C_50_N_5_H_5_)–Li and (Mg@C_50_N_5_H_5_)–Na.

Comparing (Mg@C_50_N_5_H_5_)–K and (Mg@C_50_N_5_H_5_)–Na, the former has a higher NPA charge on the Mg atom (0.744 *vs.* 0.021|*e*|) and a shorter *L*_2_ distance (2.325 *vs.* 3.496 Å) than the latter, which is theoretically beneficial for electrostatic stabilization. Nevertheless, two unfavorable factors counteract this advantage in (Mg@C_50_N_5_H_5_)–K. First, its considerably long Mg–K distance (*L*_1_, 7.474 Å) may significantly weaken both the alkali metal-cage interaction and the alkali metal-alkaline earth metal interaction, in sharp contrast to the shorter Mg–Na distance (*L*_1_, 5.841 Å) in (Mg@C_50_N_5_H_5_)–Na. Second, the compressed *L*_2_ distance can induce stronger repulsive interaction between the Mg atom and the C_50_N_5_H_5_ cage for (Mg@C_50_N_5_H_5_)–K than for (Mg@C_50_N_5_H_5_)–Na. These destabilizing factors collectively result in an even smaller |*E*_int_| value for (Mg@C_50_N_5_H_5_)–K compared with (Mg@C_50_N_5_H_5_)–Na, rendering the former thermodynamically less stable than the latter. Overall, (Mg@C_50_N_5_H_5_)–K exhibits the smallest |*E*_int_| magnitude and narrowest *ε*_gap_ value among all nine species of (M′@C_50_N_5_H_5_)–M (M = Li, Na and K; M′ = Be, Mg and Ca). Subsequently, it has the poorest thermodynamic and chemical stability throughout the studied systems.

The VIEs of (^+^M@C_50_N_5_H_5_)M′^−^ (M, M′ = Li, Na and K)^19^ range from 3.46 to 4.13 eV. So, our (M′@C_50_N_5_H_5_)–M (M = Li, Na and K; M′ = Be, Mg and Ca) with VIEs of 4.755–6.396 eV exhibit considerably higher electronic stability than their alkali analogues. For relevant alkaline-earth-based compounds, the VIEs are, respectively, in the ranges of 2.412–3.374 eV, 3.5–4.7 eV, and 2.77–3.26 eV for reported (M–HMHC)–M′,^11,27^ M^+^(calix[4]pyrrole)M′^−^,^20^ and M^+^(NH_3_)_6_M′^−^ (M = Li, Na and K; M′ = Be, Mg and Ca).^21^ Then, (M′@C_50_N_5_H_5_)–M (M = Li, Na and K; M′ = Be, Mg and Ca) also have considerably higher electronic stabilities than them.

The *ε*_gap_ values are in the range of 2.750–3.758 eV for (M–HMHC)–M′ and within the ranges of 3.22–5.63 eV, 3.55–5.03 eV and 2.66–3.18 eV for M(HF)_3_M (AM = Li and Na),^10^ M^+^(calix[4]pyrrole)M′^−^ and M^+^(NH_3_)_6_M′^−^ (M = Li, Na and K; M′ = Be, Mg and Ca),^20,21^ respectively. So, the chemical stability of our (M′@C_50_N_5_H_5_)–M (M = Li, Na and K; M′ = Be, Mg and Ca) with *ε*_gap_ values of 1.048–3.481 eV is comparable.

The *E*_int_ values range from −27.35 to −29.90 kcal mol^−1^ for the series of (Be@C_50_N_5_H_5_)–M, from −13.45 to −22.17 kcal mol^−1^ for the series of (Mg@C_50_N_5_H_5_)–M, and from −50.40 to −58.08 kcal mol^−1^ for the series of (Mg@C_50_N_5_H_5_)–M. The latter are comparable to those of reported (M–HMHC)–M′(−38.86–56.38 kcal mol^−1^).^11,27^ The *E*_int_ values are in the range of −26.60–38.02 kcal mol^−1^ for their alkali analogues of (^+^M@C_50_N_5_H_5_)M′^−^ (M, M′ = Li, Na and K).^19^ Then, our (M′@C_50_N_5_H_5_)–M (M = Li, Na and K; M′ = Be, Mg and Ca) holds good thermodynamic stability.

Comparison of the series of (Be@C_50_N_5_H_5_)–M, (Mg@C_50_N_5_H_5_)–M, and (Ca@C_50_N_5_H_5_)–M shows that, along with increasing atomic number of the alkaline earth metal atom, the *E*_int_ value first decreases and then significantly increases, which indicates that the thermodynamic stability first drops and then rises. This may be related to the changes in the NPA charges.

Therefore, the thermodynamic stability of our (M′@C_50_N_5_H_5_)–M (M = Li, Na and K; M′ = Be, Mg and Ca) is not only better than the alkalide analogues of (M@C_50_N_5_H_5_)–M′ (M, M′ = Li, Na and K),^19^ but also has good comparability with many similar alkalides.^9,11,20,21,27^

**Fig. 2:**
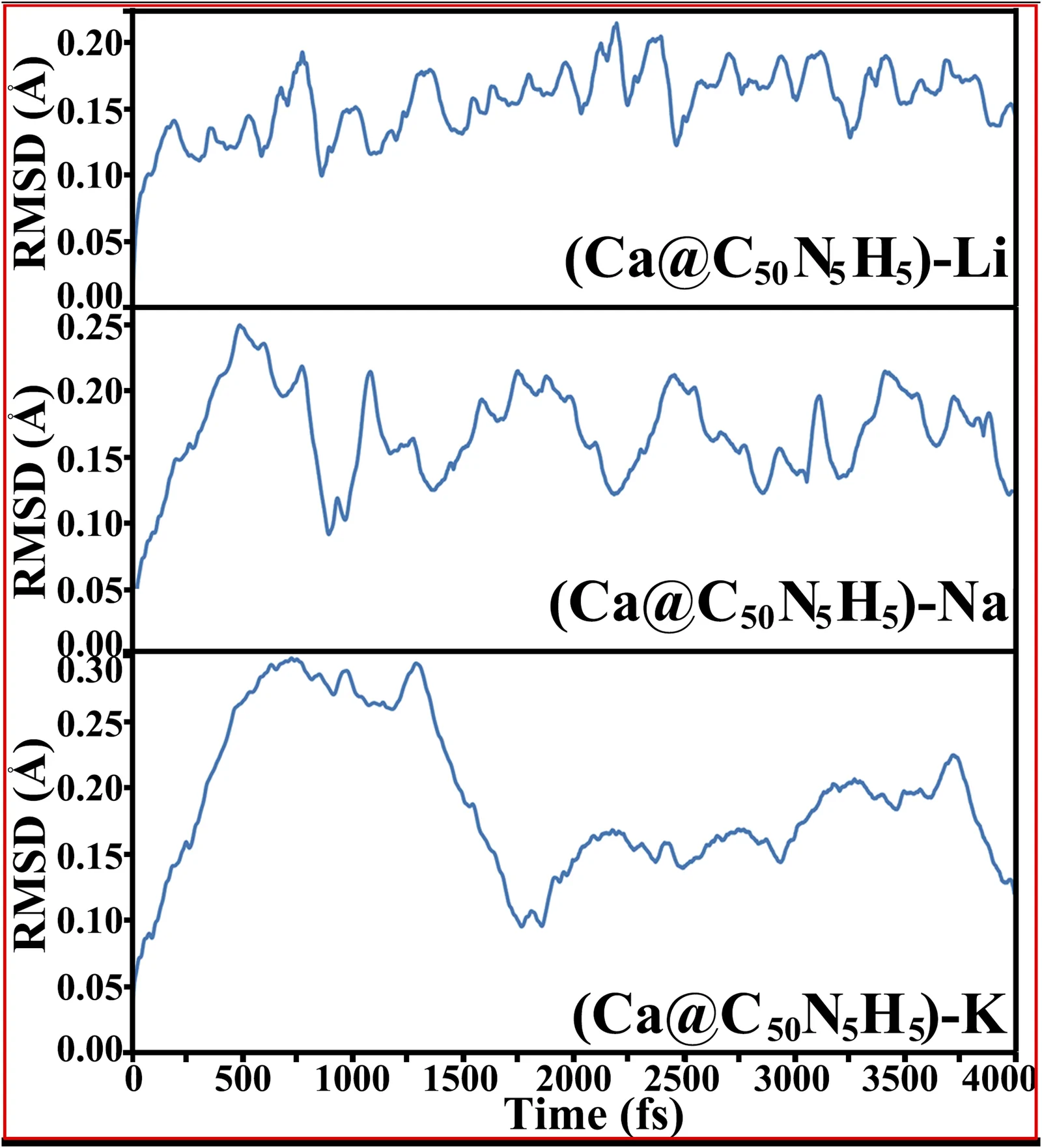
*Ab initio* molecular dynamics study for (Ca@C_50_N_5_H_5_)–M (M = Li, Na, and K): the root-mean-square deviation (RMSD) of trajectories.

Fig. 2 provides the results of AIMD simulations based on quantum mechanics. To qualitatively describe the molecular deformation due to thermal fluctuations, for each trajectory, we aligned each structure (at each time t) to the reference structure (at time 0) to eliminate translation and rotation, and then averaged deviations of all atoms in the molecule. Thus, the root mean square deviation (RMSD) at each time was obtained. As shown in Fig. 2, the RMSD associated with each trajectory was bounded within about or less than 0.3 Å and revealed no signs of dissociation or fragmentation of such molecules, demonstrating their thermodynamic stability.

### Binding characteristics

The NPA charges of M (*Q*_M_) and M′ (*Q*_M′_) in (M′@C_50_N_5_H_5_)–M (M = Li, Na and K; M′ = Be, Mg and Ca) are listed in Fig. 1. Both *Q*_M′_ and *Q*_M_ exhibit gradual trends. From the series of (Be@C_50_N_5_H_5_)–M to (Mg@C_50_N_5_H_5_)–M and then to (Ca@C_50_N_5_H_5_)–M, *Q*_M′_ gradually changes from small negative values (>−0.1|*e*|) to small positive values (<0.1|*e*|) and then to larger positive values (>0.7|*e*|), and *Q*_M_ changes from small positive values (<0.8|*e*| for M′ = Be and Mg) to larger positive values (>1.0|*e*| for M′ = Ca).

The *Q*_M_ and *Q*_M′_ for the series of (Be@C_50_N_5_H_5_)–M are very small positive and negative values, respectively. So, they are weakly electropositive and electronegative, respectively. The NPA charge values of Be in them are in the range of −0.019–0.036|*e*|. The NPA charges range from −0.28 to −0.47|*e*| for (M^+^@C_50_N_5_H_5_)M′^−^ (M, M′ = Li, Na and K).^19^ The reported weak superalkalide is a *C*_2v_ OLi_3_–Li–Li_3_O with *Q*_Na_ = −0.12|*e*| to the best of our knowledge.^17^ So, (Be@C_50_N_5_H_5_)–M (M = Li, Na and K) exhibit considerably weak alkaline-earthide behavior. Their salt-like structures may be (Be^*δ*−^@C_50_N_5_H_5_)^(^*^δ^*^′−^*^δ^*^)−^M^*δ*′+^.

**Fig. 3:**
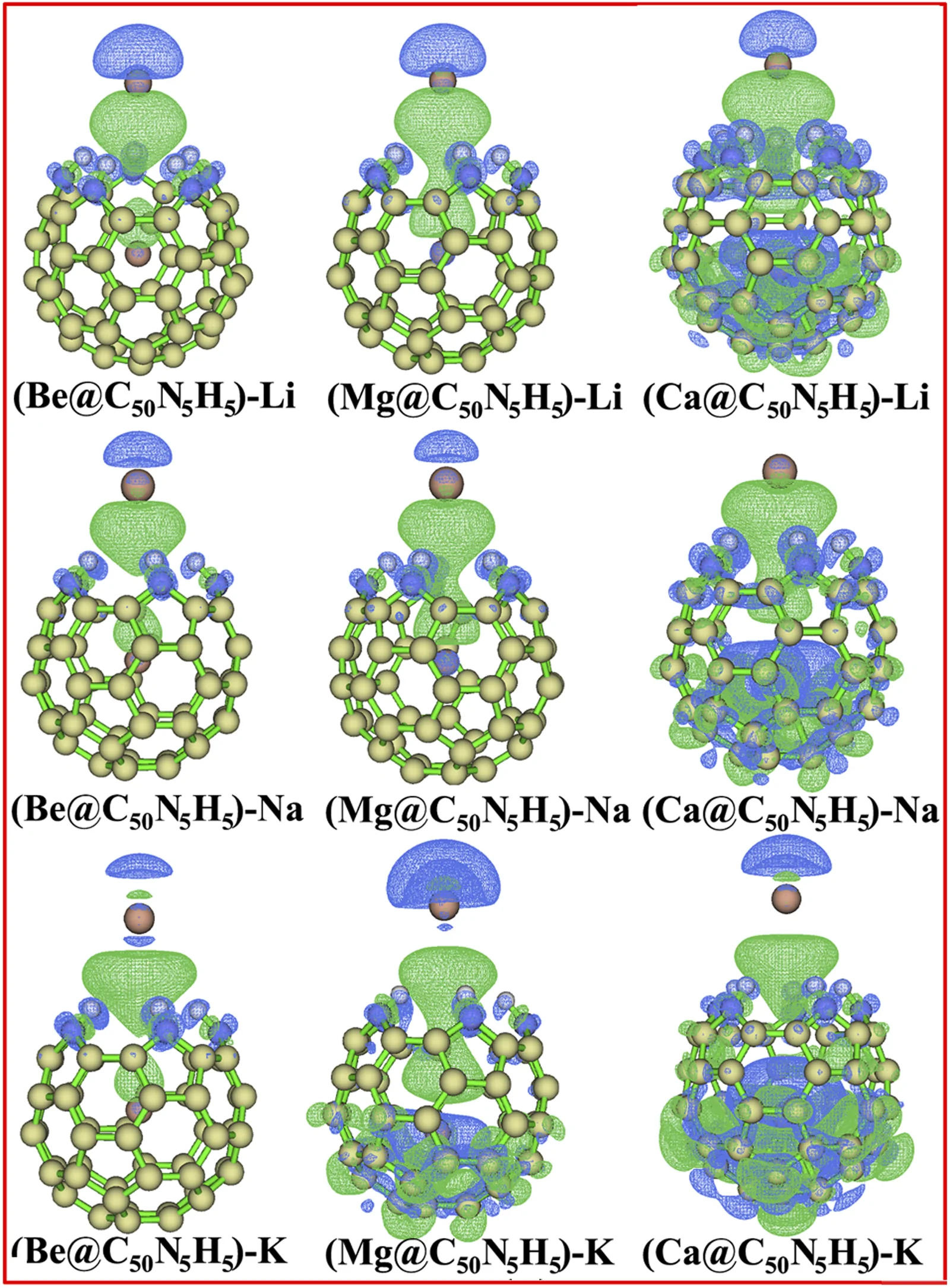
Electron density difference (EDD) maps for (M′@C_50_N_5_H_5_)–M (M = Li, Na and K; M′ = Be, Mg and Ca).

The electron density difference (EDD) maps, suggested by Lu *et al.*,^46^ are shown in Fig. 3. These blue and green isosurfaces indicate the decrease and increase in electron density in the regions, respectively. A small blue lobe is observed over the alkali atom (M = Li, Na, and K) and a small green lobe over the Be atom for the series of (Be@C_50_N_5_H_5_)–M. This electron density may provide support for the above NPA charges of M and Be in the series of (Be^*δ*−^@C_50_N_5_H_5_)^(^*^δ^*^′−^*^δ^*^)−^–M^*δ*′+^. Also, a very small blue isosurface is observed on the Mg atom for both (Mg@C_50_N_5_H_5_)–Li and (Mg@C_50_N_5_H_5_)–Na, which indicates a small positive NPA charge on the Mg atom. For (Mg@C_50_N_5_H_5_)–K and (Ca@C_50_N_5_H_5_)–M (M = Li, Na and K), a large blue isosurface exists on the alkaline earth metal atom (Mg or Ca), which provides support for the large positive NPA charge of the Mg atom in them. The salt-like structures of (Mg@C_50_N_5_H_5_)–M (M = Li and Na) with fractional oxidation number or fractional valence may be (Mg^*δ*+^@C_50_N_5_H_5_)^(^*^δ^*^′^*^+δ^*^)−^–M^*δ*′+^, while those of (Mg@C_50_N_5_H_5_)–K and (Ca@C_50_N_5_H_5_)–M are, respectively, (Mg^+^@C_50_N_5_H_5_^2−^)–K^+^ and (Ca^2+^@C_50_N_5_H_5_^(2+*δ*)+^)–M^*δ*+^.

In summary, the results of the NPA charges for the alkaline earth metal atoms show that, as the atomic number increases, there is an evolution from a very small negative charge to a very large positive charge for the alkaline earth metal atom in (M′@C_50_N_5_H_5_)–M (M = Li, Na and K; M′ = Be, Mg and Ca), and the structure changes from the weak alkaline-earthide of (Be^*δ*−^@C_50_N_5_H_5_)^(^*^δ^*^′−)−^–M^*δ*+^ to the dual-alkali-metal salts of (Mg^*δ*+^@C_50_N_5_H_5_)^(^*^δ^*^′^*^+δ^*^)−^–M^*δ*′+^ and (Ca^2+^@C_50_N_5_H_5_^(2+*δ*)+^)–M^*δ*+^ (M = Li, Na and K) as the alkali and alkaline earth metal increases. More importantly, the NPA charge and EDD maps indicate that there is a charge transfer between the metal atoms and the C_50_N_5_H_5_ cage and that the interaction between them should be primarily electrostatic.

### Static first hyperpolarizabilities

In Table 1, the alkali and alkaline earth metal atomic number dependences are shown for (M′@C_50_N_5_H_5_)–M (M = Li, Na and K; M′ = Be, Mg and Ca). As the atomic number of M increases, the *µ*_0_ value increases monotonically in each series of (Be@C_50_N_5_H_5_)–M, (Mg@C_50_N_5_H_5_)–M, and (Ca@C_50_N_5_H_5_)–M (M = Li, Na and K) but decreases monotonically in each series of (M′@C_50_N_5_H_5_)–Li, (M′@C_50_N_5_H_5_)–Na, and (M′@C_50_N_5_H_5_)–K along with the increase in the atomic number of M′ (M′ = Be, Mg and Ca). Then, (Li@C_50_N_5_H_5_)–K has the largest *µ*_0_ value (16.02 D) among the nine species. In addition, all (M′@C_50_N_5_H_5_)–M (M = Li, Na and K; M′ = Be, Mg and Ca) have downward directions of *µ*_0_ or *µ*_*z*_.

**Table 1:** Dipole moment (*µ*_0_), static first hyperpolarizability (*β*^e^_0_) and its *z*-components (*β*^e^_*zzz*_), the projection of *β* onto the dipole moment vector (*β*_prj_) hyper Rayleigh scattering response (*β*_HRS_), the sum-over-states estimation values (*β*^SOS^_0_), dipole moment between the ground state and the crucial excited state (Δ*µ*), oscillator strength (*f*_0_), and transition energy (Δ*E*) of the crucial excited state for the chair forms of (M′–@C_50_N_5_H_5_)–M (M = Li, Na and K; M′ = Be, Mg and Ca)

	*µ*_0_ (D)	*β*_HRS_ (a.u.)	*β*_prj_ (a.u.)	*β* ^e^_0_ (a.u.)	*β* ^e^_*zzz*_ (a.u.)	*β* ^SOS^_0_ (au)	Δ*µ*_*z*_ (au)	Δ*µ*_0_ (au)	*f*_0_^*z*^	*f*_0_	Δ*E* (eV)	Major contribution^a^
**(Li@C**_**50**_ **N**_**5**_ **H**_**5**_ **)–Li**												
				(9.23 × 10^3^)^b^		2.18 × 10^4^	3.587	3.637	0.182	0.182	1.230	H → L + 3
							1.123	2.025	0.000	0.145	2.440	H − 1 → L + 1
							−2.423	3.460	0.304	0.307	2.471	H → L + 7
							−0.277	1.558	0.000	0.200	3.841	H − 2 → L + 3

**(Be@C**_**50**_ **N**_**5**_ **H**_**5**_ **)–M (M′ = Be)**												
M = Li	13.02	2.33 × 10^4^	−3.51 × 10^4^	5.84 × 10^4^	−5.43 × 10^4^	1.34 × 10^5^	−1.067	1.815	0.364	0.364	2.040	H(α) → L(α) + 1
							−2.038	4.629	0.000	0.061	2.313	H(α) → L(α) + 4
							−0.865	4.691	0.000	0.112	3.826	H(β) → L(β) + 4
M = Na	13.13	1.53 × 10^4^	−2.41 × 10^4^	4.02 × 10^4^	−3.33 × 10^4^	1.23 × 10^5^	2.009	1.718	0.349	0.350	2.203	H(α) → L(α) + 1
							−0.348	5.525	0.095	0.095	2.437	H(β) → L(β) + 4
							−1.153	4.339	0.000	0.062	3.794	H(α) → L(α) + 3
							−1.591	4.342	0.000	0.066	3.838	H(α) − 2 → L(α) + 3
M = K	16.02	2.42 × 10^4^	−3.94 × 10^4^	6.56 × 10^4^	−4.55 × 10^4^	5.73 × 10^4^	0.603	2.524	0.383	0.383	1.514	H(α) → L(α) + 1
							3.854	4.462	0.081	0.081	2.022	H(α) → L(α) + 4
							−0.767	5.828	0.000	0.104	3.815	H(α) − 2 → L(α) + 4

**(Mg@C**_**50**_ **N**_**5**_ **H**_**5**_ **)–M (M′ = Mg)**												
M = Li	11.52	2.69 × 10^4^	−6.64 × 10^4^	6.64 × 10^4^	−6.35 × 10^4^	6.29 × 10^4^	−0.612	5.274	0.045	0.045	1.796	H(α) → L(α) + 1
							−1.694	1.767	0.317	0.317	2.078	H(α) − 1 → L(α) + 1
							−3.750	4.356	0.000	0.045	2.318	H(α) − 1 → L(α) + 4
							−1.211	4.396	0.000	0.106	3.813	H(β) − 2 → L(β)
M = Na	11.77	2.58 × 10^4^	−6.65 × 10^4^	6.65 × 10^4^	−5.80 × 10^4^	6.48 × 10^4^	0.396	4.909	0.035	0.035	1.846	H(α) → L(α) + 1
							−1.851	6.538	0.349	0.349	2.257	H(α) − 1 → L(α) + 1
							−2.058	4.184	0.000	0.087	2.752	H(α) → L(α) + 4
							0.828	4.204	0.000	0.139	3.822	H(β) − 3 → L(β) + 5
M = K	15.98	8.59 × 10^4^	−2.20 × 10^5^	2.20 × 10^5^	−1.93 × 10^5^	3.54 × 10^5^	1.961	5.803	0.083	0.083	0.669	H(β) → L(β) + 1
							8.985	8.071	0.000	0.064	2.128	H(β) → L(β) + 4
							8.137	9.771	0.165	0.166	2.304	H(β) → L(β) + 5
							8.036	8.105	0.000	0.064	3.809	H(β) → L(β) + 11

**(Ca@C**_**50**_ **N**_**5**_ **H**_**5**_ **)–M (M′ = Ca)**												
M = Li	5.09	2.96 × 10^5^	−7.01 × 10^5^	7.10 × 10^5^	−6.82 × 10^5^	1.03 × 10^5^	−0.732	4.167	0.031	0.031	0.958	H(β) → L(β) + 3
							3.479	5.016	0.176	0.176	1.794	H(α) − 1 → L(α) + 4
							−0.882	4.515	0.128	0.127	2.312	H(β) → L(β) + 5
							1.537	2.777	0.019	0.130	3.758	H(β) − 2 → L(β) + 4
M = Na	5.17	7.40 × 10^4^	−1.29 × 10^5^	1.46 × 10^5^	−2.44 × 10^4^	8.53 × 10^4^	−0.791	4.059	0.040	0.040	0.981	H(β) → L(β) + 3
							6.311	4.409	0.123	0.123	1.875	H(α) − 1 → L(α) + 1
							−1.401	4.760	0.279	0.279	2.882	H(α) − 1 → L(α) + 5
							1.207	2.688	0.009	0.121	3.762	H(β) → L(β) + 4
M = K	11.58	1.63 × 10^5^	−4.05 × 10^5^	4.05 × 10^5^	−3.83 × 10^5^	7.43 × 10^5^	4.460	5.772	0.073	0.073	0.861	H(α) − 1 → L(α)
							−4.340	7.713	0.000	0.091	1.987	H(α) − 1 → L(α) + 4
							−3.663	6.088	0.142	0.142	2.437	H(α) − 1 → L(α) + 6
							−2.199	7.865	0.002	0.054	3.634	H(β) → L(β) + 6

a H, L, H(α), H(β), L(α), and L(β) are HOMO, LUMO, HOMO(α), HOMO(β), LUMO(α), and LUMO(β), respectively.

b The value in parentheses is from ref. 17.

The *β*_HRS_ and *β*_prj_, as well as *β*^e^_0_, are also shown in Table 1. The main components of the polarizability (*α*, au) and hyperpolarizability (*β*, au) tensors are shown in Table S2. Overall, the alkali and alkaline earth metal atomic number dependences are also shown for *β*_HRS_ and *β*_prj_ as well as *β*^e^_0_. As the atomic number of M (M = Li, Na, K) increases, the *β*_HRS_, *β*_prj_, and *β*^e^_0_ values undergo an initial decrease and a subsequent increase in each series of (Be@C_50_N_5_H_5_)–M and (Ca@C_50_N_5_H_5_)–M (M = Li, Na and K). In the (Mg@C_50_N_5_H_5_)–M series, the *β*_HRS_ value presents the aforementioned variation tendency, while the *β*_prj_ and *β*^e^_0_ values rise in a monotonic manner. It can be found that, for the (Be@C_50_N_5_H_5_)–M series, which feature long *L*_2_ distances and weak alkaline-earthide characteristics, the variation orders of *β*_HRS_, *β*_prj_ and *β*^e^_0_ are consistent with the trend of the NPA charge of alkaline earth metal atom (*Q*_M′_) as the atomic number of M (M = Li, Na, K) increases, while the NLO ordering follows the variation trend of the *L*_2_ distances with increasing atomic number of M (M = Li, Na and K) for the (Ca@C_50_N_5_H_5_)–M species, characterized by short *L*_2_ distances and dual-alkali-metal salt-like features. In contrast, for the (Mg@C_50_N_5_H_5_)–M series with hybrid salt-like features and both long and short *L*_2_ distances, the orders of *β*_prj_ and *β*^e^_0_ coincide with the trend of *Q*_M′_, whereas the *β*_HRS_ order follows the NPA charge variation of the alkali metal atom (*Q*_M_) as the atomic number of M (M = Li, Na and K) increases. For each series of (M′@C_50_N_5_H_5_)–Li, (M′@C_50_N_5_H_5_)–Na and (M′@C_50_N_5_H_5_)–K (M′ = Be, Mg and Ca), *β*_HRS_, *β*_prj_, and *β*^e^_0_ values all increase monotonically with the increase in atomic number of M′. These *β* orders exhibit an inverse correlation with the sequences of *Q*_M′_ and band gap values (*ε*_gap_) within their respective series. Then, (Ca@C_50_N_5_H_5_)–K has the largest absolute values of *β*_HRS_, *β*_prj_, and *β*^e^_0_ among the nine species. These values are 1.63 × 10^5^, −4.05 × 10^5^, and 4.05 × 10^5^ au for *β*_HRS_, *β*_prj_, and *β*^e^_0_, respectively. All (M′@C_50_N_5_H_5_)–M (M = Li, Na and K; M′ = Be, Mg and Ca) have downward directions of *β*_prj_ or *β*_*zzz*_. The main components of the polarizability (*α*, au) and hyperpolarizability (*β*, au) tensors are shown in Table S2.

The *β*^e^_0_ values of our species are in the range of 4.02 × 10^4^–4.05 × 10^5^ au. For the reported (M^+^@C_50_N_5_H_5_)–M′^−^ (M, M′ = Li, Na and K),^19^ the *β*^e^_0_ values are in the range of 5.86 × 10^3^–1.91 × 10^5^ au. So, our (M′@C_50_N_5_H_5_)–M (M = Li, Na and K; M′ = Be, Mg and Ca) have better NLO responses than their alkali analogues. For the relative alkalides, the *β*_0_ values of M^+^(calix[4]pyrrole)M′^−^ and M^+^(NH_3_)_6_M′^−^ (M = Li, Na and K; M′ = Be, Mg and Ca)^20,21^ are, respectively, in the range of 3.80 × 10^3^–1.90 × 10^5^ a.u. and 2.1 × 10^3^–6.4 × 10^4^ a.u. So, our (M′@C_50_N_5_H_5_)–M (M = Li, Na and K; M′ = Be, Mg and Ca) also have better NLO responses than those of M^+^(calix[4]pyrrole)M′^−^.

**Fig. 4:**
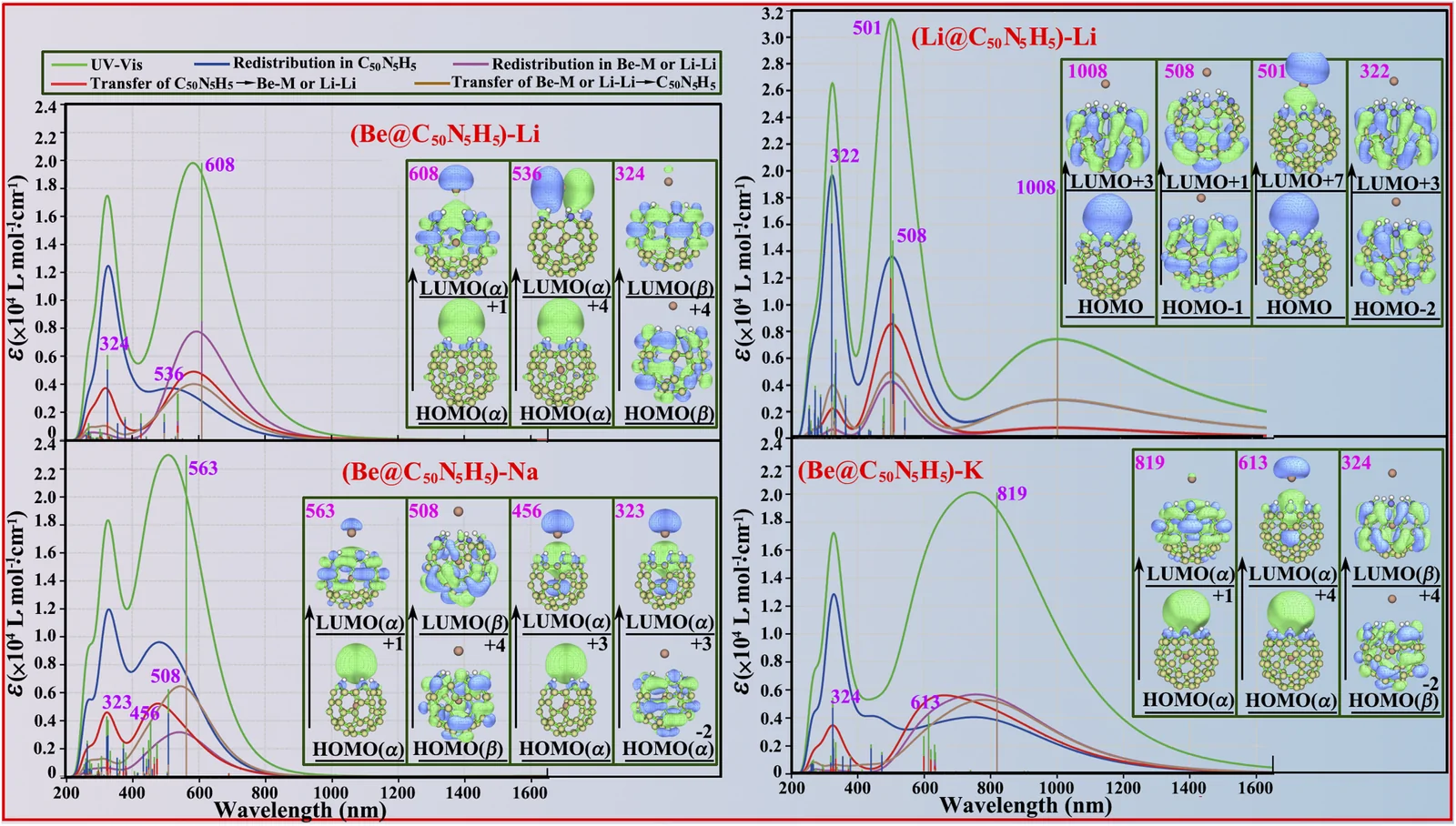
Charge-transfer spectra of (Be@C_50_N_5_H_5_)–M (M = Li, Na and K) and (Li@C_50_N_5_H_5_)–Li. The molecular orbitals at an isovalue of 0.02 a.u.

**Fig. 5:**
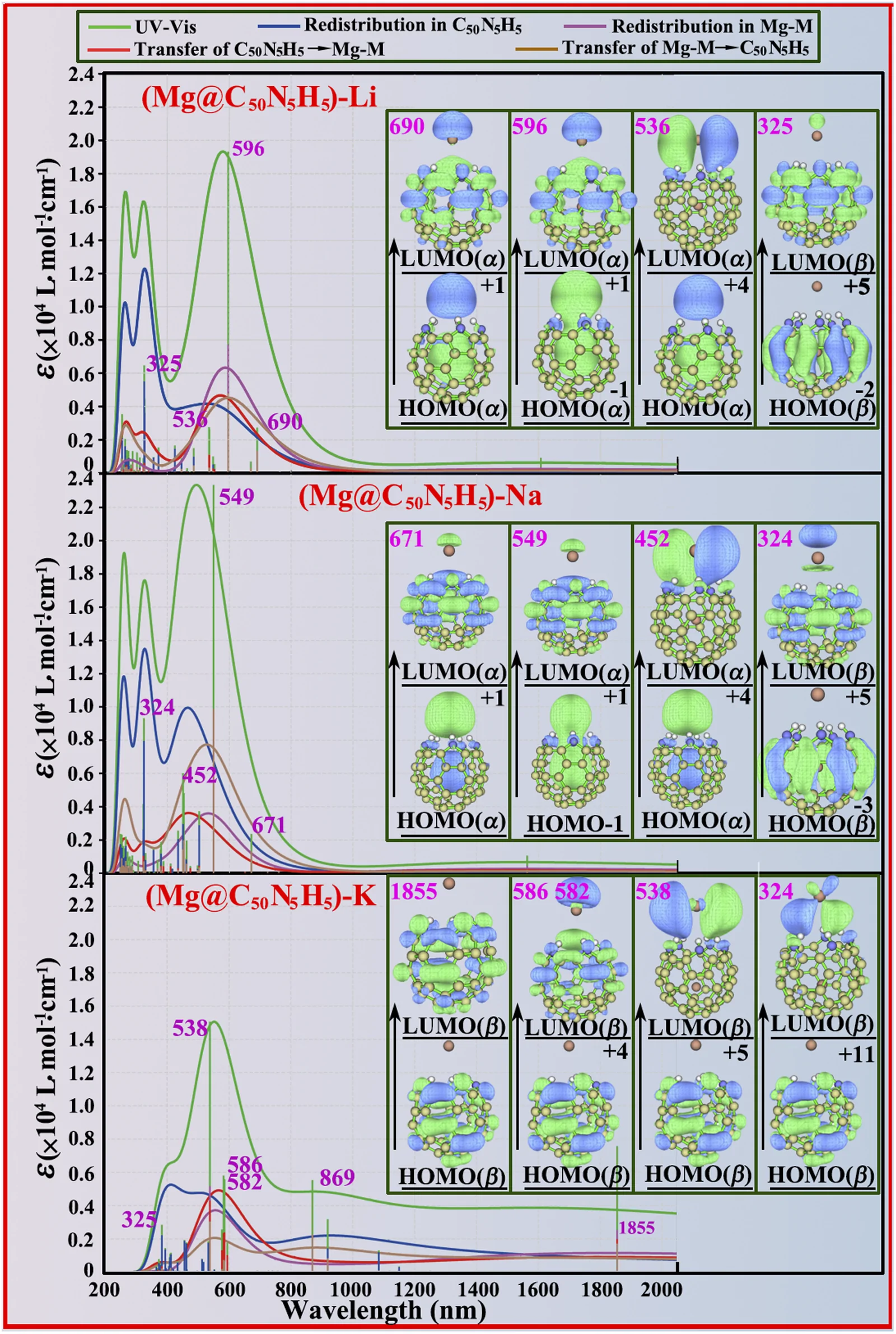
Charge-transfer spectra of (Mg@C_50_N_5_H_5_)–M (M = Li, Na and K). The molecular orbitals at an isovalue of 0.02 a.u.

**Fig. 6:**
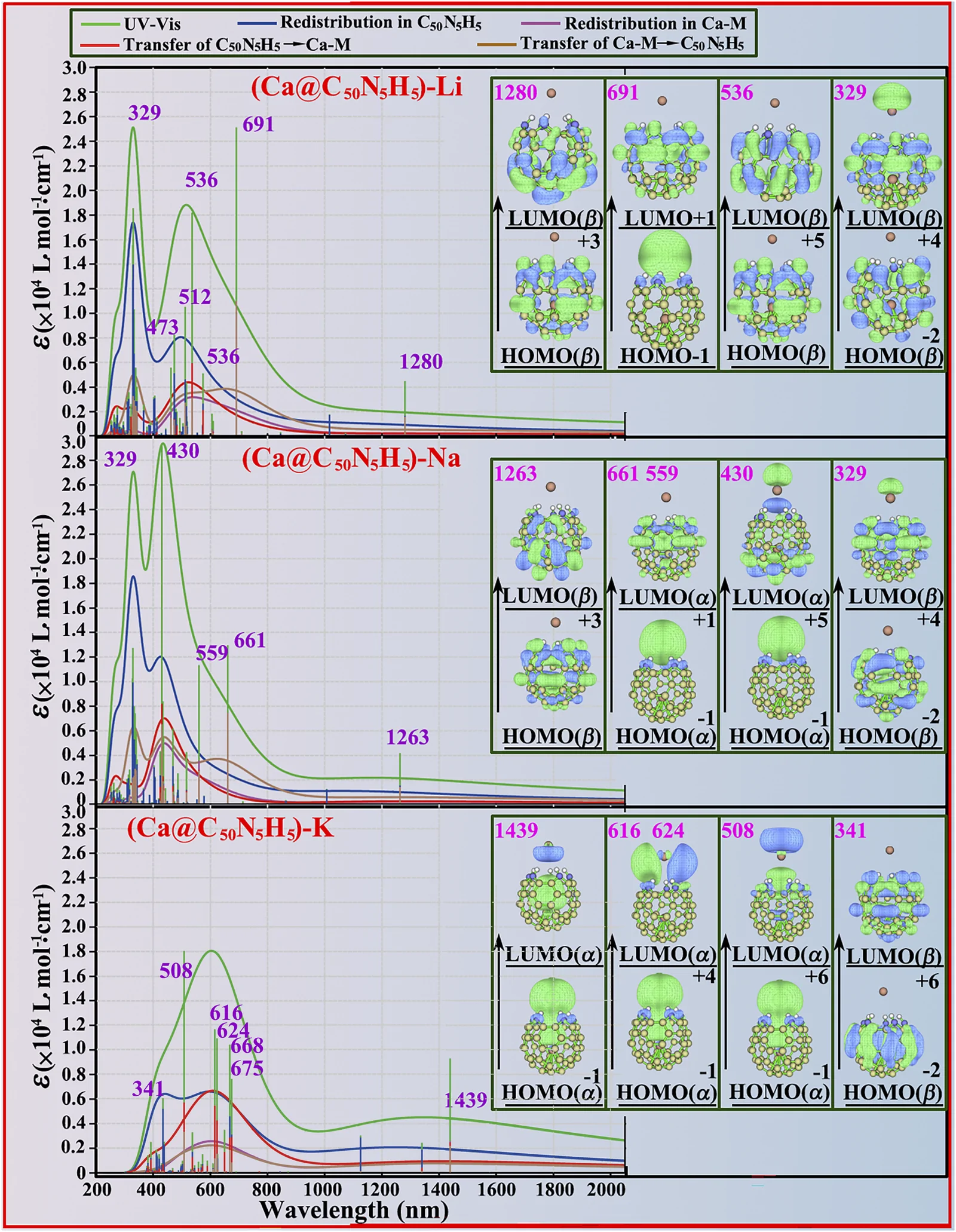
Charge-transfer spectra of (Ca@C_50_N_5_H_5_)–M (M = Li, Na and K). The molecular orbitals at an isovalue of 0.02 a.u.

The *β*^e^_0_ value can be estimated by using the sum-over-states estimation:

6 *β*^e^_0_ ∝ (Δ*µ*·*f*_0_)/Δ*E*^3^

where Δ*E*, *f*_0_, and Δ*µ* are, respectively, the transition energy, oscillator strength, and the difference in transition dipole moment between the ground state and the crucial excited state. The estimated first hyperpolarizabilities (*β*^SOS^_0_) are listed in Table 1. Obviously, the difference between *β*^SOS^_0_ and *β*^e^_0_ is significant. So, given that the *β*^SOS^_0_ results cannot be directly used to explain the *β*^e^_0_ results, we turned our attention to the Δ*E* and the *z*-components of *f*_0_ and Δ*µ*. The charge-transfer spectra of (M′@C_50_N_5_H_5_)–M (M = Li, Na and K; M′ = Be, Mg and Ca) are shown in Fig. 4–6.

From Fig. 4–6, each spectrum exhibits a broad peak in the low-energy region and one or two sharp peaks in the high-energy region. For the sharp peak(s) in the high-energy region, the wavelengths are 323–325 nm. From Table 1, it can be found that the *z*-component of *f*_0_ is 0.000 (*f*_0_^*z*^) for the high-energy sharp peaks. So, it has nearly no contribution to *β*^e^_*zzz*_. Thus, it is the low-energy broad peak that makes the key contribution to *β*^e^_*zzz*_. For these low-energy broad peaks, it can be found that the wavelength first decreases and then increases in each series of (Be@C_50_N_5_H_5_)–M, (Mg@C_50_N_5_H_5_)–M, and (Ca@C_50_N_5_H_5_)–M (M = Li, Na and K) as the atomic number of M increases. The longer the wavelength, the smaller the Δ*E*. Considering that *β*^e^_0_ is proportional to the reciprocal of Δ*E*'s cube, the order of the wavelength of the broad peak may explain the order of the *β*^e^_0_ for each series of (Be@C_50_N_5_H_5_)–M, (Mg@C_50_N_5_H_5_)–M, and (Ca@C_50_N_5_H_5_)–M (M = Li, Na and K).

In addition, for the (Be@C_50_N_5_H_5_)–M (M = Li, Na, and K) series, both Δ*µ*_0_ and *f*_0_^*z*^ of the first excited state initially decrease and then increase with the rising atomic number of M, which follows the variation trends of the *β*_HRS_, *β*_prj_, and *β*^e^_0_ as well as that of the above *Q*_M′_. For the (Mg@C_50_N_5_H_5_)–M series, the |Δ*µ*_0_^*z*^| of the excited state with the maximum oscillator strength (*f*_0_) increases monotonically as the atomic number of M increases, showing the sequence of 1.694 (M = Li, *f*_0_ = 0.317) < 1.851 (M = Na, *f*_0_ = 0.349) < 8.137 (M = K, *f*_0_ = 0.165); this trend is consistent with the variations of *β*_prj_, *β*^e^_0_ and above *Q*_M′_. For the (Ca@C_50_N_5_H_5_)–M series, the |Δ*µ*_0_^*z*^| of the maximum-*f*_0_ excite state exhibits a trend of first decreasing and then increasing with the increase of M atomic number, with a |Δ*µ*_0_^*z*^| order of 3.479 (M = Li, *f*_0_ = 0.176) < 1.401 (M = Na, *f*_0_ = 0.279) < 3.663 (M = K, *f*_0_ = 0.142), which well matches the variation patterns of *β*_prj_, *β*^e^_0_ and above *L*_2_ distance.

Considering the charge transfer, Fig. 4–6 show that the low-energy broad peak mainly belongs to redistribution in M′–M and transfer between M′–M and C_50_N_5_H_5_ for each case of (M′@C_50_N_5_H_5_)–M (M = Li, Na and K; M′ = Be, Mg and Ca). The orbital transition mainly responds to s → p or p → p transition in the *z*-axis (HOMO(α) → LUMO(α) + 1 ((Be@C_50_N_5_H_5_)–M)), or HOMO(α) − 1 → LUMO(α) + 1 or LUMO(α) + 5 or LUMO(α) + 6 ((Mg@C_50_N_5_H_5_)–M (M = Li or Na) and (Ca@C_50_N_5_H_5_)–M (M = Li or Na or K)), and HOMO(β) → LUMO(β) + 5 (Mg@C_50_N_5_H_5_)–K) for all the nine species. For M′@C_50_N_5_H_5_)–K (M′ = Be, Mg and Ca) in their respective series, *i.e.*, (Be@C_50_N_5_H_5_)–M, (Mg@C_50_N_5_H_5_)–M, and (Ca@C_50_N_5_H_5_)–M (M = Li, Na and K), there exist some shoulder absorptions with large *f*_0_. In addition, there is a low-energy absorption with wavelength > 1000 nm for (Mg@C_50_N_5_H_5_)–K and (Ca@C_50_N_5_H_5_)–M (M = Li, Na and K), with a wavelength that first decreases and then increases in series (Ca@C_50_N_5_H_5_)–M (M = Li, Na and K) as the atomic number of M increases. This may be the reason why M′@C_50_N_5_H_5_)–K (M′ = Be, Mg and Ca) has the largest *β*^e^_0_ value in their respective series.

Comparing the CTS maps of (Be@C_50_N_5_H_5_)–Li and (Li@C_50_N_5_H_5_)–Li (see Fig. 4), it can be observed that it mainly involves the s → p transition from Be–Li to the C_50_N_5_H_5_ cage, which corresponds to the characteristic absorption peak (*λ*_max_) for (Be@C_50_N_5_H_5_)–Li, but mainly involves the s → p transition around the outside Li atom, which corresponds to *λ*_max_ for (Li@C_50_N_5_H_5_)–Li. More importantly, the *λ*_max_ is 107 nm larger in wavelength for (Be@C_50_N_5_H_5_)–Li than for (Li@C_50_N_5_H_5_)–Li, which means the Δ*E* of the former is smaller than that of the latter. This may be the most important reason why the *β*^e^_0_ value of (Be@C_50_N_5_H_5_)–Li with co-doping of alkali and alkaline earth metal elements is slightly larger than that of (Li@C_50_N_5_H_5_)–Li with single-element doping, although the latter holds a significant low-energy absorption at 1008 nm that cannot be ignored.

It has been^43^ suggested that the vibrational first hyperpolarizability tensor (*β*^nr^_*zzz*_) is also important for the total hyperpolarizability. The Raman frequency is required for the calculation of *β*^nr^_*zzz*_. So, the *β*^nr^_*zzz*_ of (M′–@C_50_N_5_H_5_)–M (M = Li, Na and K; M′ = Be, Mg and Ca) were obtained at the M06-2X/6-31G(d) level. Table 3 also gives the *β*^e^_*zzz*_ at the same level.

Comparing Tables 1 and 2, the *β*^e^_*zzz*_ obtained at the M06-2X/6-31G(d) and M06-2X/6-311++G(d,p) levels are, respectively, −5.93 × 10^3^ and −2.62 × 10^5^ a.u., which indicates that the M06-2X/6-31G(d) method significantly underestimated the *β*^e^_*zzz*_ value. Still, from Table 3, it can be found that, numerically, *β*^nr^_*zzz*_ is an order of magnitude smaller than *β*^e^_*zzz*_ at the same level. All the *η* values are very small (<0.20) except that of (Ca@C_50_N_5_H_5_)–M. So, it is not *β*^nr^_*zzz*_ but *β*^e^_*zzz*_ that makes the pivotal contribution to the total *β*_0_ of each of (M′–@C_50_N_5_H_5_)–M (M = Li, Na and K; M′ = Be, Mg and Ca).

**Table 2:** Electronic and vibrational first hyperpolarizability tensors (*β*^e^_*zzz*_ and *β*^nr^_*zzz*_) and ratio *η* of *β*^nr^_*zzz*_/*β*^e^_*zzz*_ for (M′–@C_50_N_5_H_5_)–M (M = Li, Na and K; M′ = Be, Mg and Ca) at the M06-2X/6-31G(d) level

	(M′–@C_50_N_5_H_5_)–M		
	*β* ^e^_*zzz*_ (a.u.)	*β* ^nr^_*zzz*_ (a.u.)	*H*
**(Be@C**_**50**_ **N**_**5**_ **H**_**5**_ **)–M (M′ = Be)**			
M = Li	−1.36 × 10^4^	−1.33 × 10^3^	0.10
M = Na	−1.69 × 10^4^	−1.90 × 10^3^	0.11
M = K	−1.07 × 10^4^	−1.42 × 10^3^	0.01

**(Mg@C**_**50**_ **N**_**5**_ **H**_**5**_ **)–M (M′ = Mg)**			
M = Li	−2.66 × 10^4^	−2.64 × 10^3^	0.10
M = Na	−3.26 × 10^4^	−3.01 × 10^3^	0.09
M = K	−1.29 × 10^5^	−6.91 × 10^3^	0.05

**(Ca@C**_**50**_ **N**_**5**_ **H**_**5**_ **)–M (M′ = Ca)**			
M = Li	−2.70 × 10^4^	−4.69 × 10^3^	0.17
M = Na	−3.34 × 10^4^	−4.50 × 10^3^	0.13
M = K	−5.93 × 10^3^	−1.01× 10^4^	1.80

**Table 3:** *β*_HRS_ and *β*_prj_ of the chair forms at different incident laser frequencies (*ω*)

	*ω* = 1907 nm		*ω* = 1460 nm		*ω* = 1340 nm		*ω*= 1064 nm	
	*β*_HRS_ (a.u.)	*β*_prj_ (a.u.)	*β*_HRS_ (a.u.)	*β*_prj_ (a.u.)	*β*_HRS_ (a.u.)	*β*_prj_ (a.u.)	*β*_HRS_ (a.u.)	*β*_prj_ (a.u.)
**(Be@C**_**50**_ **N**_**5**_ **H**_**5**_ **)–M (M′ = Be)**								
M = Li	2.92 × 10^4^	7.31 × 10^4^	3.76 × 10^5^	9.15 × 10^5^	9.16 × 10^4^	−2.13 × 10^5^	2.05 × 10^5^	−5.87 × 10^5^
M = Na	5.68 × 10^3^	1.36 × 10^4^	2.66 × 10^4^	7.22 × 10^4^	4.91 × 10^5^	1.19 × 10^6^	1.76 × 10^5^	4.75 × 10^5^
M = K	6.50 × 10^4^	1.74 × 10^5^	1.15 × 10^5^	3.17 × 10^5^	3.47 × 10^5^	9.37 × 10^5^	1.26 × 10^5^	−3.39 × 10^5^

**(Mg@C**_**50**_ **N**_**5**_ **H**_**5**_ **)–M (M′ = Mg)**								
M = Li	9.84 × 10^5^	−2.37 × 10^6^	2.51 × 10^4^	6.30 × 10^4^	3.78 × 10^5^	9.20 × 10^5^	1.64 × 10^4^	−4.31 × 10^4^
M = Na	2.60 × 10^7^	−6.28 × 10^7^	9.54 × 10^3^	2.88 × 10^3^	4.67 × 10^4^	−8.17 × 10^4^	3.87 × 10^6^	9.33 × 10^6^
M = K	8.64 × 10^4^	2.25 × 10^5^	5.44 × 10^4^	1.52 × 10^5^	2.73 × 10^5^	7.59 × 10^5^	2.22 × 10^5^	−5.76 × 10^5^

**(Ca@C**_**50**_ **N**_**5**_ **H**_**5**_ **)–M (M′ = Ca)**								
M = Li	8.29 × 10^5^	−1.99 × 10^6^	3.06 × 10^4^	−7.36 × 10^4^	4.23 × 10^4^	1.06 × 10^5^	1.03 × 10^5^	2.66 × 10^5^
M = Na	6.37 × 10^4^	−1.52 × 10^5^	3.07 × 10^4^	7.87 × 10^4^	4.98 × 10^4^	1.22 × 10^5^	4.72 × 10^5^	−1.10 × 10^6^
M = K	2.03 × 10^6^	4.64 × 10^6^	1.46 × 10^5^	3.85 × 10^5^	6.07 × 10^5^	5.93 × 10^5^	2.01 × 10^5^	−5.40 × 10^5^

### Dynamic first hyperpolarizabilities

For (M′–@C_50_N_5_H_5_)–M (M = Li, Na and K; M′ = Be, Mg and Ca), the *β*_HRS_ and *β*_prj_ with the frequency dispersion at wavelengths (*ω*) of 1907, 1460, 1340, and 1064 nm are listed in Table 3. Their tensors are shown in Table S2.

With increasing atomic number of M (M = Li, Na, K), the *β*_HRS_ and *|β*_prj_| of (Be@C_50_N_5_H_5_)–M exhibit three distinct variation trends. At *ω* = 1907 nm and 1460 nm, they first decrease and then rise markedly. At *ω* = 1340 nm, they increase significantly at first and then decrease, while they display a monotonic decreasing trend at *ω* = 1064 nm. Similarly, the *β*_HRS_ and |*β*_prj_| of (Ca@C_50_N_5_H_5_)–M also present three distinct variation trends, whereas only two trends are observed for (Mg@C_50_N_5_H_5_)–M. For the (Mg@C_50_N_5_H_5_)–M series, *β*_HRS_ and |*β*_prj_| increase significantly first and then decrease at *ω* = 1907 nm and 1604 nm, while they exhibit the opposite tendency of initial decrease followed by a subsequent increase at *ω* = 1460 nm and 1340 nm. In terms of (Ca@C_50_N_5_H_5_)–M, its *β*_HRS_ and |*β*_prj_| values decrease significantly at first and then rise at *ω* = 1907 nm and present an initial increase followed by a decrease at *ω* = 1640 nm. In contrast, a monotonic increasing behavior is observed at *ω* = 1460 nm and 1340 nm.

With the rise in atomic number of M, *β*_HRS_ and |*β*_prj_| display two variation trends for each (M′@C_50_N_5_H_5_)–Li and (M′@C_50_N_5_H_5_)–Na series, while three distinct trends are observed for (M′@C_50_N_5_H_5_)–K (M′ = Be, Mg and Ca). For the (M′@C_50_N_5_H_5_)–Li and (M′@C_50_N_5_H_5_)–Na series, *β*_HRS_ and |*β*_prj_| first increase significantly and then decrease at *ω* = 1907 nm, whereas they exhibit the opposite tendency of initial decrease followed by a subsequent increase at *ω* = 1460 nm. (M′@C_50_N_5_H_5_)–Li at *ω* = 1340 nm and (M′@C_50_N_5_H_5_)–Na at *ω* = 1064 nm, these parameters show an initial increase followed by a significant decrease. Conversely, they first decrease significantly and then increase for (M′@C_50_N_5_H_5_)–Na at *ω* = 1340 nm and (M′@C_50_N_5_H_5_)–Li at *ω* = 1064 nm. For the (M′@C_50_N_5_H_5_)–K series, *β*_HRS_ and |*β*_prj_| values decrease significantly first and then rise at *ω* = 1460 nm and 1340 nm, while they present a slight decrease after an initial increase at *ω* = 1064 nm. In addition, a monotonic increasing behavior is observed at *ω* = 1907 nm. As a result, all (M′@C_50_N_5_H_5_)–Li, (M′@C_50_N_5_H_5_)–Na, and (M′@C_50_N_5_H_5_)–K (M′ = Be, Mg and Ca) exhibit a consistent trend of initial decrease followed by a subsequent increase at *ω* = 1460 nm.

## Conclusions

To compare the NLO performances of hybrid alkali–alkaline-earth metal doping and dual-alkali-metal doping, we theoretically constructed a series of (M′@C_50_N_5_H_5_)–M (M = Li, Na and K; M′ = Be, Mg and Ca), in which an alkaline-earth metal atom is encapsulated inside the C_50_N_5_H_5_ cage and an alkali metal atom is anchored on the cage exterior.

Along with the increase in atomic number of the doped metal atoms, the structure changes from a weak alkaline-earthide of (Be^*δ*−^@C_50_N_5_H_5_)^(^*^δ^*^′−^*^δ^*^)−^–M^*δ*′+^ to a dual-alkali-metal salt of (Mg^*δ*+^@C_50_N_5_H_5_)^(^*^δ^*^′^*^+δ^*^)−^–M^*δ*′+^ and (Ca^2+^@C_50_N_5_H_5_^(2+*δ*)+^)–M^*δ*+^ (M = Li, Na and K). Analysis of VIE and *ε*_gap_ results shows that our (M′@C_50_N_5_H_5_)–M possesses improved stability relative to their all-alkali alkalide analogues, (M@C_50_N_5_H_5_)M, with stability performance comparable to that of most reported alkalide systems.

It is noteworthy that our species also exhibit higher NLO responses than their alkali analogues. With increasing atomic number of M (M = Li, Na, K), the *β*^e^_0_ presents an initial decrease followed by a subsequent increase for the (Be@C_50_N_5_H_5_)–M and (Ca@C_50_N_5_H_5_)–M series, while a monotonic increasing trend is observed for the (Mg@C_50_N_5_H_5_)–M series. A similar trend is exhibited for the dynamic first hyperpolarizability.

This work reveals the potential of rationally designed cage-confined hybrid alkali–alkaline-earth metal-doped systems for NLO applications. We hope this study can motivate experimental efforts toward the synthesis and characterization of such emerging alkaline-earthide and dual-alkali-metal salt candidates for advanced NLO materials.

## Conflicts of interest

There are no conflicts to declare.

## Supplementary Material

RA-016-D6RA04746G-s001

## Data Availability

The data will be available on request. Supplementary information (SI): data of optimized geometries, computational details on *β*^nr^_*ijk*_ and *β*^e^_*ijk*_ of (M′@C_50_N_5_H_5_)–M (M = Li, Na and K; M′ = Be, Mg and Ca). See DOI: https://doi.org/10.1039/d6ra04746g.
