# Supplementary material for: Alkali and alkaline-earth metal hybrid doping enhances NLO response: from a weak alkaline-earthide of (Beδ−@C50N5H5)(δ′−δ)−‒Mδ′+ to dual-alkali-metal salts of (Mgδ+@C50N5H5)(δ+δ′)−–Mδ'+ and (Ca2+@C50N5H5)(2+δ)−–Mδ+
Source: RSC Adv. 2026 Aug 18;16(41):48564–75. doi: 10.1039/d6ra04746g (PMC13482749; doi:10.1039/d6ra04746g)
Supplement: Supplementary file 1 [file RA-016-D6RA04746G-s001.pdf]

Supplementary Information (SI) for RSC Advances.
This journal is © The Royal Society of Chemistry 2026

Supporting Information

Alkali and alkaline earth metal hybrid doping brings better NLO response: from weak alkaline-earthide of ($Be^{\delta-}$@$C_{50}N_5H_5$)-$M^{\delta+}$ to dual-alkali-metal salt of ($Mg^{\delta+}$@$C_{50}N_5H_5$)-$M^{\delta+}$ and (Ca^{2+}@$C_{50}N_5H_5^{(2+\delta)+}$)-$M^{\delta+}$

Feng Xu,[a] Zhi-Xuan Wang,[b] Rui-Wen Liu,[a] Hao-Chen,[a] Jin-Jin Wei,[a] Zhijun Wang,[a] Wen Zhang,[a] Hong-Liang Xu,[c] Yin-Feng Wang,[*a]

[a] *Key Laboratory of Jiangxi Province for Special Optoelectronic Artificial Crystal Materials, Key Laboratory of Energy Conversion Optoelectronic Functional Materials of Jiangxi Education Institutes, School of Chemistry and Chemical Engineering. Jinggangshan University, Ji'an, Jiangxi 343009 (P.R. China). E-mail: cyclont@yeah.net*

[b] *College of Chemistry, Huazhong Agricultural University, Wuhan, 430070 (PR China).*

[c] *Institute of Functional Material Chemistry, National & Local United Engineering Laboratory for Power Batteries, Department of Chemistry, Northeast Normal University, Changchun 130024 (P.R. China).*

Contents

Optimized Cartesian coordinates S3
Computational details on β_{ijk}^{nr} and β_{ijk}^{e}..S16
Table S1. Dipole moment (μ_0), static first hyperpolarizability (β_0^e), and its Hyper Rayleigh Scattering response (β_{HRS}) at M06-2X/6-311++G(d,p) level...............S18
Table S2. The main components of the polarizability and hyperpolarizability tensor..S19
Fig. S1. Fractional orbital density (FOD) plot at σ = 0.003 e/Bohr^3 (TPSS/def2-TZVP (T =5000 K) level) for (M'@$C_{50}N_5H_5$)-M (M = Li, Na & K; M' = Be, Mg & Ca) (FOD depicted in pink)..S21

Optimized Cartesian coordinates

($Be@C_{50}N_5H_5$)-Li

C	0.28303032	-2.95783054	1.97148235
C	-1.09537918	-2.76149422	1.97218714
C	0.93109683	-3.29789899	0.74501070
C	2.20485201	-2.62380267	0.74414037
C	2.28968779	-1.89591490	1.97008589
C	2.90250699	-0.64574325	1.96940831
C	-2.96533012	0.18998383	1.97231647
C	-2.72612949	-1.18162725	1.97274962
C	-2.84892850	-1.90399125	0.74667097
C	-1.81395104	-2.90688433	0.74642495
C	-1.18398369	-3.27090657	-0.45011106
C	0.22323094	-3.47147321	-0.45087109
C	2.74472348	-2.13671492	-0.45265015
C	1.98980751	-2.27996325	-1.68816996
C	0.76473038	-2.92849216	-1.68730465
C	-0.34196519	-2.39205185	-2.45599053
C	-1.55362040	-2.59824069	-1.68613331
C	-2.54906124	-1.63349971	-1.68595863
C	-3.23269268	-1.28541487	-0.44967751
C	-3.47701993	0.11488446	-0.45006884
C	-3.32539702	0.82750756	0.74593771
C	-0.96507413	3.34166510	-0.45225867
C	-0.24043360	3.41857117	0.74364776
C	1.18629332	3.21535137	0.74289505
C	1.85937062	2.93952175	-0.45374725
C	1.10064456	2.81819286	-1.68924689
C	-1.13144563	2.13404800	-2.45715332
C	-2.34133676	1.91805533	-1.68714889
C	-2.22128703	2.67667806	-0.45148688
C	-2.69114044	2.12160072	0.74523338
C	-1.96600689	2.22867337	1.97108615
C	-0.73542456	2.87991711	1.97035623

C	1.51232526	2.55969602	1.96921687
C	2.51223995	1.59086847	1.96889834
C	3.17729449	1.28582479	0.74225006
C	2.88004116	1.95026889	-0.45407761
C	2.78211478	1.18836782	-1.68975632
C	1.67972437	1.73359910	-2.45839388
C	0.85654661	0.88462623	-3.19168367
C	-0.57886571	1.08903800	-3.19107430
C	-2.95153581	0.67337313	-1.68654067
C	-2.38118880	-0.41583723	-2.45574821
C	-1.21694353	-0.21294321	-3.19045299
C	-0.17576098	-1.22197137	-3.19044790
C	1.10564641	-0.54372642	-3.19139537
C	2.16757448	-1.06370122	-2.45778155
C	3.02007948	-0.17719232	-1.68932241
C	3.37023478	-0.86037376	-0.45342159
C	3.42505890	-0.13392121	0.74261179
C	-0.27168621	3.01353299	-1.68851534
N	-1.82224105	-1.88051598	2.79812314
N	-2.35160719	1.15393785	2.79750968
N	0.37097886	2.59426868	2.79549286
N	2.58321184	0.45107785	2.79503689
N	1.22751749	-2.31451725	2.79671932
H	1.89461061	0.33122676	3.53077221
H	0.27265532	1.90176345	3.53074571
H	-1.72209148	0.84591827	3.53146871
H	-1.33524575	-1.37832449	3.53322472
H	0.89934586	-1.69363880	3.52929953
Li	0.00367453	0.00285261	6.06928860
Be	0.00027029	0.00033258	0.29008149

$(Be@C_{50}N_5H_5)$-Na

C	1.83751469	1.72300530	-2.41920687
C	1.83780853	0.43075581	-2.93817736
C	0.61092371	2.45446694	-2.38986915
C	0.60939719	3.22070256	-1.16934792
C	1.83515184	2.93065933	-0.49556211
C	1.83296768	2.83622610	0.89382948
C	1.83400260	-2.66513352	-1.31639097
C	1.83610241	-1.77193515	-2.38477409
C	0.61050790	-1.51580229	-3.07231124
C	0.61159878	-0.11813420	-3.42356524
C	-0.58444043	0.61013371	-3.42511156
C	-0.58477196	1.92913241	-2.89507311

C	-0.58796353	3.44556251	-0.47886621
C	-1.82330092	2.86441478	-0.98149864
C	-1.82179765	2.12754950	-2.15561079
C	-2.59128678	0.90150056	-2.24512319
C	-1.82121159	-0.04532005	-3.02867015
C	-1.82239149	-1.38973449	-2.69092118
C	-0.58659606	-2.15646055	-2.72975326
C	-0.58876179	-3.06815800	-1.63911614
C	0.60633551	-3.29403483	-0.94514568
C	-0.59475314	-2.50607237	2.41103355
C	0.60113880	-1.91797955	2.84102274
C	0.60084532	-0.58075673	3.37827046
C	-0.59535218	0.14089181	3.47456208
C	-1.83078777	-0.45453848	2.98919185
C	-2.59805017	-2.04503536	1.28001992
C	-1.82796771	-2.98549041	0.48886200
C	-0.59330741	-3.26179171	1.20704736
C	0.60403233	-3.39180054	0.49265118
C	1.83036226	-2.81921046	0.94948478
C	1.82902881	-2.07908904	2.12913038
C	1.82855128	0.02855872	2.97564202
C	1.82951473	1.37914569	2.63616072
C	0.60287570	2.10811549	2.70232684
C	-0.59429596	1.51948336	3.12799398
C	-1.82903013	1.81645892	2.41825866
C	-2.59855294	0.58979482	2.33858676
C	-3.33078229	0.30192095	1.19102690
C	-3.33050914	-1.04343994	0.65048868
C	-1.82576493	-2.89143103	-0.89407117
C	-2.59363603	-1.85244754	-1.55310981
C	-3.32825716	-0.94506926	-0.79606227
C	-3.32719533	0.46110280	-1.14949947
C	-3.32871634	1.23182962	0.07862655
C	-2.59447271	2.41092830	0.15997941
C	-1.82704846	2.70554651	1.35474357
C	-0.59021790	3.34913002	0.93933360
C	0.60501265	3.03259868	1.59679516
C	-1.83045297	-1.74076024	2.47235766
N	2.66203811	-0.64055343	-2.53955888
N	2.65746652	-2.61851083	-0.17368674
N	2.65390839	-0.97917727	2.43819653
N	2.65649503	2.01215254	1.68676311
N	2.66136909	2.22150047	-1.39019151
H	3.40280836	1.48426309	1.24684472

H	3.40193632	-0.72369260	1.80236727
H	3.40192616	-1.93091909	-0.12570216
H	3.40514987	-0.47272618	-1.86953226
H	3.40661797	1.63891289	-1.02363617
Na	5.96869296	-0.00223719	0.00462598
Be	0.15374987	0.00023851	0.00114581

($Be@C_{50}N_5H_5$)-K

C	1.68254135	-0.18063747	-2.96441700
C	1.68422209	-1.51156387	-2.55538125
C	0.45579717	0.40528239	-3.40220598
C	0.45314725	1.76873673	-2.93536490
C	1.67835516	1.96776298	-2.22881939
C	1.67579322	2.76830363	-1.08964489
C	1.68333902	-2.89727299	0.65226303
C	1.68480415	-2.87529755	-0.73997324
C	0.45965209	-3.11083682	-1.43524856
C	0.45928484	-2.24533053	-2.58765872
C	-0.73769060	-1.68077269	-3.04634905
C	-0.73940320	-0.32246617	-3.46393238
C	-0.74484915	2.37676277	-2.53923517
C	-1.97966742	1.60721850	-2.56375453
C	-1.97707307	0.29594129	-3.01299255
C	-2.74549510	-0.71407598	-2.31127614
C	-1.97412741	-1.94214009	-2.32492725
C	-1.97359984	-2.77433783	-1.21642438
C	-0.73698572	-3.39428785	-0.76476760
C	-0.73844841	-3.41685402	0.65604710
C	0.45686806	-3.15479537	1.33747421
C	-0.74618211	-0.43205949	3.45088232
C	0.44908570	0.29714426	3.41477819
C	0.44725913	1.67478249	2.99150126
C	-0.74993079	2.29514560	2.61243257
C	-1.98478531	1.52536834	2.60999305
C	-2.75007510	-0.78682760	2.28257592
C	-1.97854172	-2.01459035	2.25870567
C	-0.74363684	-1.77646990	2.99067741
C	0.45421784	-2.32615165	2.51653405
C	1.67922736	-1.59177962	2.50991914
C	1.67673270	-0.27460545	2.96112747
C	1.67382915	1.89585187	2.29381361
C	1.67339552	2.73196870	1.18054140
C	0.44652993	3.33998311	0.77425143
C	-0.75028849	3.14856693	1.47626658

C	-1.98528699	2.93130322	0.73787290
C	-2.75365492	1.92717502	1.44793116
C	-3.48579169	0.98079256	0.73735727
C	-3.48362848	-0.40486800	1.16339411
C	-1.97606866	-2.81149839	1.12462996
C	-2.74495155	-2.41930797	-0.04079933
C	-3.48127038	-1.23837622	-0.02287128
C	-3.48156667	-0.36775794	-1.18218603
C	-3.48434881	1.00377422	-0.71233055
C	-2.75079278	1.97206449	-1.39108833
C	-1.98396347	2.95326705	-0.64809636
C	-0.74752103	3.19379984	-1.37675800
C	0.44800923	3.36309511	-0.66673549
C	-1.98289322	0.20045482	3.01726886
N	2.51135818	-2.09251120	-1.57216112
N	2.50823369	-2.14109118	1.51024956
N	2.50234438	0.77533414	2.50898546
N	2.50194009	2.62714915	0.04433552
N	2.50721106	0.85454244	-2.47779865
H	3.23453087	1.92412926	0.03382759
H	3.23565979	0.56980232	1.83750849
H	3.23801940	-1.56269300	1.10545197
H	3.24095440	-1.52753593	-1.14847161
H	3.23960476	0.62806759	-1.81212755
K	6.23819007	0.00275907	0.00185075
Be	-0.00235606	-0.00141514	0.00084233

($Mg@C_{50}N_5H_5$)-Li

C	-2.12860783	-2.07735105	1.96808808
C	-2.84202279	-0.87864275	1.96758840
C	-1.98563456	-2.79544720	0.74092932
C	-0.66001094	-3.36471153	0.74065312
C	-0.04046858	-2.97392504	1.96761523
C	1.32003907	-2.66590987	1.96677754
C	-1.71457326	2.43299216	1.96481278
C	-2.63435015	1.38424811	1.96596377
C	-3.27252045	1.02522799	0.73876320
C	-3.40440152	-0.41127080	0.73985170
C	-3.29191731	-1.13212206	-0.45688653
C	-2.56489931	-2.35346328	-0.45633010
C	0.05897123	-3.48039991	-0.45690188
C	-0.52682995	-2.98263069	-1.69353349
C	-1.80092683	-2.43529956	-1.69318669
C	-2.07767711	-1.23745018	-2.46355853

C	-2.99874025	-0.42241372	-1.69406696
C	-2.87206292	0.95843417	-1.69514374
C	-3.03088608	1.71156788	-0.45910278
C	-2.09376311	2.78015234	-0.46022735
C	-1.44344697	3.11088291	0.73646528
C	1.99734383	2.84974175	-0.46238859
C	2.51264482	2.33475006	0.73494287
C	3.25050904	1.09514826	0.73543004
C	3.45776522	0.39581093	-0.46147021
C	2.88600994	0.90974494	-1.69804952
C	0.95222612	2.21808206	-2.46753630
C	0.02472139	3.02575776	-1.69797858
C	0.69142491	3.41070454	-0.46207699
C	-0.03646399	3.42947131	0.73554481
C	0.50183876	2.93454883	1.96331916
C	1.78366178	2.38423320	1.96303288
C	2.94611418	0.43163059	1.96392448
C	2.81861058	-0.95750516	1.96497070
C	2.99692075	-1.66735339	0.73761554
C	3.32788842	-1.01949612	-0.46036152
C	2.67198392	-1.42276395	-1.69630515
C	2.40469675	-0.22273066	-2.46652533
C	1.22685638	-0.11517169	-3.20085921
C	0.48522165	1.13112294	-3.20143593
C	-1.32774652	2.71961636	-1.69709118
C	-1.81798489	1.59099157	-2.46564349
C	-0.92926874	0.81091434	-3.20032258
C	-1.06185787	-0.63325391	-3.19932510
C	0.27070695	-1.20562163	-3.19957915
C	0.53216985	-2.35838360	-2.46403597
C	1.75775942	-2.46540642	-1.69507851
C	1.44524763	-3.16662563	-0.45782151
C	2.04576668	-2.75191340	0.73881788
C	2.17685944	2.10138420	-1.69851738
N	-2.61605903	0.24220711	2.79280143
N	-0.57897255	2.56524734	2.79040528
N	2.26070547	1.34629588	2.78984727
N	1.97804590	-1.73068445	2.79204770
N	-1.03582684	-2.41269809	2.79402010
H	1.45283846	-1.26908790	3.52759840
H	1.66053671	0.98939373	3.52638095
H	-0.42416812	1.88308756	3.52581634
H	-1.91973786	0.17888610	3.52839863
H	-0.75886377	-1.76819519	3.52752764

Li	0.00369783	0.00443739	6.06691884
Mg	0.00013915	0.0017211	0.2939225

(Mg@$C_{50}N_5H_5$)-Na

C	-1.82628821	0.99327387	2.80876833
C	-1.82646683	-0.39450925	2.95323587
C	-0.59787079	1.70393338	2.97685605
C	-0.59894314	2.77721219	2.01293283
C	-1.82797431	2.68490137	1.28967971
C	-1.82981783	2.97682117	-0.07478661
C	-1.82983361	-2.92798788	0.53879255
C	-1.82826956	-2.36210741	1.81416180
C	-0.59917342	-2.30389249	2.54097882
C	-0.59805534	-1.05535665	3.26381647
C	0.59967614	-0.35471902	3.46154854
C	0.59978463	1.05889035	3.31466876
C	0.59773025	3.18324762	1.40655187
C	1.83467197	2.48471051	1.72662525
C	1.83557684	1.45303504	2.65315514
C	2.60468896	0.24927855	2.39990623
C	1.83556073	-0.87661241	2.89535085
C	1.83438189	-2.07664473	2.20058768
C	0.59746137	-2.82594396	2.03102512
C	0.59578692	-3.40262954	0.73203441
C	-0.60245254	-3.42923705	0.00534221
C	0.59120232	-1.74836047	-3.01009249
C	-0.60636586	-1.06400595	-3.25906394
C	-0.60626704	0.37092899	-3.40815793
C	0.59129937	1.09190444	-3.30533161
C	1.82815556	0.38473714	-3.00476619
C	2.59901121	-1.61497450	-1.80058401
C	1.82984122	-2.73690422	-1.29614977
C	0.59231761	-2.80561705	-2.06035945
C	-0.60428267	-3.12779267	-1.40537697
C	-1.83277170	-2.45311926	-1.68450451
C	-1.83369344	-1.41487308	-2.61670058
C	-1.83360664	0.84644958	-2.85154336
C	-1.83246452	2.05402704	-2.15258241
C	-0.60403635	2.77180582	-2.01835459
C	0.59242733	2.32192305	-2.59325303
C	1.83007645	2.41181492	-1.83128441
C	2.59920721	1.21012234	-2.09419555
C	3.33535014	0.61760198	-1.07180865

C	3.33522347	-0.82495815	-0.92192181
C	1.83164482	-3.02684651	0.05982502
C	2.60256463	-2.20883038	0.97695893
C	3.33710094	-1.12818327	0.49632375
C	3.33817230	0.12698256	1.22293962
C	3.33711865	1.20598885	0.25378708
C	2.60265549	2.36234301	0.50190223
C	1.83172414	2.97437093	-0.56388058
C	0.59600858	3.48039738	0.01676941
C	-0.60232835	3.35732509	-0.69986129
C	1.82813986	-0.99447643	-2.86145113
N	-2.65241943	-1.31775728	2.28040134
N	-2.65618288	-2.57257832	-0.54653132
N	-2.65861448	-0.27164819	-2.61306597
N	-2.65609901	2.40539734	-1.06381552
N	-2.65206608	1.75843227	1.96028788
H	-3.40524851	1.78072671	-0.78547903
H	-3.40571050	-0.20080896	-1.93054501
H	-3.40580973	-1.90452637	-0.40296890
H	-3.40341461	-0.97565152	1.69066138
H	-3.40218985	1.30194592	1.45261251
Na	-5.99992055	0.00093872	0.00425094
Mg	-0.15936837	0.00019986	-0.00025486

(Mg@$C_{50}N_5H_5$)-K

C	-1.82145415	-1.58278937	2.41615933
C	-1.87702870	-2.50798859	1.37560480
C	-0.58414687	-1.38349582	3.09990059
C	-0.50174184	0.02098040	3.41581305
C	-1.68805417	0.62784718	2.91233028
C	-1.61687659	1.91049434	2.36701957
C	-1.82292563	-2.01145636	-2.07370072
C	-1.87814286	-2.72341816	-0.87665192
C	-0.69857591	-3.36647596	-0.40144223
C	-0.69800893	-3.22947635	1.03004118
C	0.51232558	-3.06421520	1.73004966
C	0.56958274	-2.11461046	2.78478322
C	0.74003124	0.67782285	3.40560381
C	1.93401311	-0.05837547	3.04413945
C	1.85709627	-1.42977100	2.73557248
C	2.59150967	-1.96547993	1.62859324
C	1.75486070	-2.97039310	0.98125571
C	1.75493391	-3.10246829	-0.40263944

C	0.51144687	-3.33613791	-1.12059083
C	0.56831435	-2.60348890	-2.33565442
C	-0.58525920	-1.94355931	-2.78265672
C	0.81575159	1.41543094	-3.16618381
C	-0.35468117	2.08137521	-2.75777239
C	-0.30562463	3.03097256	-1.67585780
C	0.90291524	3.31174057	-1.02312505
C	2.10814481	2.58228289	-1.41077123
C	2.74280206	0.38312772	-2.33816676
C	1.93183230	-0.63096695	-2.97563187
C	0.73955790	0.02406067	-3.46972913
C	-0.50347521	-0.62351725	-3.35744757
C	-1.68936108	0.06696487	-2.97687407
C	-1.61621610	1.42900782	-2.67722265
C	-1.54384862	2.92048865	-0.97302940
C	-1.54433726	3.05450255	0.41262408
C	-0.30589072	3.29518362	1.08205611
C	0.90299815	3.44650369	0.38811828
C	2.10751119	2.80220791	0.90593871
C	2.86025919	2.29543352	-0.21591781
C	3.53931145	1.05108382	-0.09661573
C	3.46890266	0.08947207	-1.17498821
C	1.85647896	-1.92053659	-2.41436685
C	2.59166242	-2.23887161	-1.22717133
C	3.39868170	-1.25510793	-0.59926662
C	3.39619832	-1.11839142	0.82799451
C	3.46926895	0.31040527	1.14194518
C	2.74467120	0.81672378	2.23006804
C	2.05426251	2.09592323	2.10740298
C	0.81563979	1.98764249	2.84792818
C	-0.35426385	2.56560827	2.32252652
C	2.05436562	1.66230615	-2.46034125
N	-2.69545321	-2.42581927	0.23154097
N	-2.56740835	-0.86813096	-2.40244477
N	-2.41042277	2.12221583	-1.74143456
N	-2.41079696	2.41866064	1.31924225
N	-2.56726597	-0.39778089	2.52301588
H	-3.19603261	1.87748562	0.97262862
H	-3.17959641	1.64081986	-1.28639260
H	-3.34210753	-0.56665130	-1.82216424
H	-3.34964734	-1.65264339	0.15791366
H	-3.32286205	-0.20624254	1.87421270
K	-6.33947391	0.09906057	-0.01084656
Mg	1.13059828	-0.13054526	-0.03812255

$(Ca@C_{50}N_5H_5)$-Li

C	-0.33151252	-2.94788174	2.01747169
C	-1.65183425	-2.47981077	2.01864667
C	0.23566782	-3.40393878	0.79216148
C	1.62075624	-3.00031795	0.79691316
C	1.84816322	-2.31382386	2.02846799
C	2.71548477	-1.21577430	2.03943669
C	-2.87930468	0.79141305	2.01684772
C	-2.92318509	-0.60482714	2.01928241
C	-3.18632647	-1.28601137	0.79119716
C	-2.37761872	-2.47652435	0.79389118
C	-1.83366814	-2.96571883	-0.41259499
C	-0.49015539	-3.43693638	-0.41279488
C	2.26523015	-2.63986486	-0.39764857
C	1.50643457	-2.62849757	-1.64447551
C	0.15326410	-3.02421859	-1.64579951
C	-0.80960825	-2.26771036	-2.40735092
C	-2.05212769	-2.22818527	-1.63836066
C	-2.83355712	-1.07324879	-1.64663217
C	-3.45084975	-0.60095971	-0.40761460
C	-3.40445900	0.81342088	-0.41023302
C	-3.09739065	1.48382576	0.78563903
C	-0.27135542	3.46204753	-0.42035167
C	0.44953893	3.38715829	0.78685878
C	1.80560315	2.89635492	0.79327185
C	2.42895552	2.49573753	-0.39935624
C	1.66920046	2.52646699	-1.64764084
C	-0.66510707	2.30620995	-2.41032468
C	-1.90732420	2.34977104	-1.64570721
C	-1.64164888	3.07246045	-0.42136364
C	-2.21605529	2.62229100	0.78570166
C	-1.49330728	2.58453178	2.01156474
C	-0.14613441	2.96915604	2.01140981
C	1.98763866	2.19707642	2.02578309
C	2.78422826	1.04769134	2.03835143
C	3.38000302	0.61467907	0.81599275
C	3.23819189	1.33177851	-0.38928393
C	2.97656923	0.60211820	-1.62194588
C	2.01993222	1.36795431	-2.41186067
C	1.04065563	0.68318291	-3.18497894
C	-0.34033469	1.17172648	-3.17234054
C	-2.76124370	1.24391523	-1.65221207

C	-2.44138053	0.07471698	-2.42800393
C	-1.23565253	0.03400114	-3.19368945
C	-0.41680364	-1.15422832	-3.17285122
C	0.99450964	-0.74969406	-3.18132991
C	1.92559290	-1.49173075	-2.40764648
C	2.93407664	-0.78627975	-1.62032927
C	3.14804730	-1.52906489	-0.38900878
C	3.33567913	-0.82199697	0.81700443
C	0.34591454	3.00623513	-1.64906076
N	-2.17610395	-1.46474320	2.83902037
N	-2.08090654	1.60639002	2.83289424
N	0.86964983	2.45189836	2.83252761
N	2.61443538	-0.07921919	2.85912160
N	0.71666176	-2.49705480	2.83588490
H	1.94080522	-0.05834974	3.62016289
H	0.63357586	1.81307763	3.58775476
H	-1.54514137	1.19315648	3.59181133
H	-1.60213086	-1.07461357	3.58254505
H	0.52648387	-1.85477611	3.60077622
Li	-0.02179891	0.00543716	6.01594870
Ca	0.03986465	-0.01038791	-0.69428271

($Ca@C_{50}N_5H_5$)-Na

C	1.88891450	-0.59517094	-2.91048004
C	1.89824397	-1.86924356	-2.32118819
C	0.65404503	-0.07939420	-3.40565268
C	0.65413673	1.33548565	-3.13585415
C	1.89093814	1.63237384	-2.48493272
C	1.90049793	2.59896906	-1.46921667
C	1.91167778	-2.78114808	1.05093661
C	1.91074786	-2.95851123	-0.33819749
C	0.67750132	-3.28423068	-0.98481606
C	0.67258520	-2.59254931	-2.24336454
C	-0.53987262	-2.10345656	-2.78035359
C	-0.54787595	-0.81200271	-3.37123679
C	-0.54374407	2.00798284	-2.83574659
C	-1.78418849	1.24667318	-2.74663192
C	-1.78258258	-0.13729067	-3.01327429
C	-2.54107313	-1.02535418	-2.17172463
C	-1.76099603	-2.25346197	-2.01825375
C	-1.76121164	-2.92142061	-0.79635981
C	-0.52060920	-3.49138719	-0.27344949
C	-0.51927990	-3.31006123	1.12715108
C	0.67893640	-2.93329514	1.76098309
C	-0.53364790	0.05582493	3.47323579

C	0.67178678	0.77135830	3.32199238
C	0.67076990	2.07382801	2.70919058
C	-0.52670667	2.65574035	2.25864025
C	-1.76955478	1.89258787	2.36085231
C	-2.52703654	-0.45187701	2.36271694
C	-1.75532696	-1.67893554	2.52172002
C	-0.53073680	-1.34221915	3.21225467
C	0.67874338	-1.95037022	2.81006031
C	1.90544514	-1.23322194	2.70346894
C	1.90189781	0.14673321	2.96264682
C	1.90437030	2.19694907	1.99658001
C	1.90829129	2.88111838	0.77408999
C	0.67802743	3.42451564	0.29268684
C	-0.52481034	3.35111093	1.02717175
C	-1.75751226	3.01236964	0.32842675
C	-2.54239552	2.12994442	1.18001815
C	-3.32300747	1.09025134	0.59645843
C	-3.30392726	-0.23723801	1.21130419
C	-1.76182680	-2.63212957	1.49924792
C	-2.54498933	-2.42579132	0.31197795
C	-3.32787761	-1.23794324	0.16008866
C	-3.31518335	-0.53552135	-1.09938494
C	-3.32217309	0.90855910	-0.82684814
C	-2.54580778	1.76428399	-1.65014459
C	-1.75982712	2.84043491	-1.04907676
C	-0.53543252	2.99086547	-1.81535444
C	0.67417412	3.24529980	-1.13125826
C	-1.76383375	0.62261661	2.96239683
N	2.71671503	-2.28858256	-1.26414876
N	2.71819759	-1.90226837	1.77909522
N	2.70975345	1.11035937	2.34676368
N	2.71750014	2.59868160	-0.33224495
N	2.69522637	0.49172493	-2.55722426
H	3.50462456	1.95314037	-0.25396639
H	3.50800112	0.83964512	1.77104437
H	3.51480990	-1.43533481	1.34396610
H	3.50545859	-1.71847891	-0.95554114
H	3.50655391	0.37523235	-1.94905223
Na	5.86395809	0.00317975	-0.01195198
Ca	-0.88484182	-0.00672458	-0.0125823

($Ca@C_{50}N_5H_5$)-K

C	1.73529955	-0.59083088	-2.91128437

C	1.74310758	-1.86482199	-2.32504136
C	0.50136979	-0.07221397	-3.40593246
C	0.50173800	1.34207718	-3.13196373
C	1.73782914	1.63777956	-2.47891450
C	1.74670053	2.60071706	-1.46166515
C	1.75183976	-2.78620111	1.04606834
C	1.75248290	-2.95945643	-0.34220153
C	0.51985115	-3.28197645	-0.99183839
C	0.51693663	-2.58745297	-2.24909588
C	-0.69422008	-2.09752151	-2.78728243
C	-0.70081705	-0.80408576	-3.37568115
C	-0.69578616	2.01492134	-2.83280467
C	-1.93695679	1.25429945	-2.74786507
C	-1.93535817	-0.12934322	-3.01799437
C	-2.69369866	-1.01945044	-2.17812113
C	-1.91619924	-2.24928146	-2.02712552
C	-1.91845877	-2.92058226	-0.80654396
C	-0.67875562	-3.49183707	-0.28307013
C	-0.67915313	-3.31402773	1.11875687
C	0.51798325	-2.93831042	1.75432284
C	-0.69527991	0.04703496	3.47353244
C	0.51054283	0.76190691	3.32490689
C	0.51095599	2.06594018	2.71492442
C	-0.68537745	2.64998498	2.26498130
C	-1.92903274	1.88799103	2.36448911
C	-2.68620800	-0.45725027	2.35908138
C	-1.91641237	-1.68583787	2.51629262
C	-0.69263936	-1.35105344	3.20905841
C	0.51685436	-1.95799294	2.80609625
C	1.74416646	-1.24104727	2.70442774
C	1.74093792	0.13690462	2.96702448
C	1.74577226	2.19155102	2.00466767
C	1.75164628	2.87747949	0.78466819
C	0.52235236	3.42188077	0.30196583
C	-0.68130282	3.34866637	1.03456609
C	-1.91338099	3.01310626	0.33329200
C	-2.69911123	2.12873276	1.18236393
C	-3.47663040	1.08979748	0.59432157
C	-3.45830404	-0.23913980	1.20571332
C	-1.92180024	-2.63672455	1.49119308
C	-2.70208666	-2.42675631	0.30253444
C	-3.48095455	-1.23679066	0.15262777
C	-3.46556423	-0.53122414	-1.10456783
C	-3.47340574	0.91162172	-0.82889775

C	-2.69874920	1.77000327	-1.65046329
C	-1.91382823	2.84438032	-1.04507682
C	-0.68818277	2.99555941	-1.80932933
C	0.52030011	3.24627071	-1.12258898
C	-1.92459346	0.61595665	2.96303251
N	2.56276024	-2.28982428	-1.26805185
N	2.56076442	-1.91134500	1.78087697
N	2.55300746	1.10412408	2.35705238
N	2.56544924	2.60013145	-0.32283219
N	2.54432729	0.49648280	-2.55657591
H	3.32844876	1.92754402	-0.24204016
H	3.33284487	0.82511179	1.76180213
H	3.33985423	-1.42844323	1.33359065
H	3.33202125	-1.70177452	-0.94669262
H	3.33590870	0.37367095	-1.92545267
K	6.33061374	0.00272727	-0.00386280
Ca	-1.01367058	0.00274871	-0.00992291

Computational details on β_{ijk}^{nr} and β_{ijk}^{e}

Using the Born-Oppenheimer approximation, β_0 can be written into an electronic contribution (β_{ijk}^{e}, $i, j, k = x, y, z$) and a vibrational contribution (β_{ijk}^{v}), and the latter can be further divided into nuclear relaxation and curvature terms (β_{ijk}^{nr} and β_{ijk}^{curv}). Then, the following equation is obtained:

$$\beta_{ijk} = \beta_{ijk}^{e} + \beta_{ijk}^{v} = \beta_{ijk}^{e} + \beta_{ijk}^{nr} + \beta_{ijk}^{curv} \tag{1}$$

β_{ijk}^{curv} is usually smaller and far more computationally expensive than β_{ijk}^{nr}. For this reason, β_{ijk}^{curv} is frequently neglected. β_{ijk}^{nr} is given in terms of vibronic energies and dipole moment matrix elements over the vibronic wave functions.[1]

According to Bishop and co-workers,[2] if we denote the equilibrium molecular geometry with an OEEF (F_i) present by R_F and without the field present, by R_0, then we may define:

$$(\Delta\mu_i)_{R_0} = \mu_i(F, R_0) - \mu_i(0, R_0) \tag{2}$$

$$(\Delta\mu_i)_{R_F} = \mu_i(F, R_F) - \mu_i(0, R_0) \tag{3}$$

β_{ijk}^{e} can be derived from the expansion of eq 5&6. And β_{ijk}^{nr} can be derived from the expansion of eq 6:

$$(\Delta\mu_i)_{R_0} = (\alpha_{ij}^{e}(0;0)) \times F_j + \frac{1}{2}(\beta_{ijk}^{e}(0;0,0)) \times F_j F_k + \cdots \tag{4}$$

$$(\Delta\mu_i)_{R_F} = (\alpha_{ij}^{e}(0;0) + \alpha_{ij}^{nr}(0;0)) \times F_j + \frac{1}{2}(\beta_{ijk}^{e}(0;0,0) + \beta_{ijk}^{nr}(0;0,0)) \times F_j F_k + \cdots \tag{5}$$

This approach is used in this paper to compute the $\beta_{zzz}^{e}(F)$ and $\beta_{zzz}^{nr}(F)$ contributions within a permanent static electric field.

References

[1] R. Zaleśny, M. Garcia-Borràs, R. W. Góra, M. Medved, J. M. Luis, *Phys. Chem. Chem. Phys.* **2016**, 18, 22467.

[2] D. M. Bishop, M. Hasan, B. Kirtman, *J. Chem. Phys.* **1995**, 103, 4157.

Table S1. Dipole moment (μ_0), static first hyperpolarizability (β_0^e), and its Hyper Rayleigh Scattering response (β_{HRS}) at M06-2X/6-311++G(d,p) level.

	μ_z (D)	μ_0 (D)	β_{HRS} (a.u.)	β_0^e (a.u.)	β_{zzz}^e (a.u.)
($Be@C_{50}N_5H_5$)-M	(M' = Be)				
M = Li	-11.19	11.19	3.73×10^3	9.88×10^3	-7.91×10^3
M = Na	-10.79	10.79	1.37×10^3	3.74×10^3	-2.14×10^3
M = K	-14.34	14.34	1.13×10^5	2.75×10^5	-2.69×10^5
($Mg@C_{50}N_5H_5$)-M	(M' = Mg)				
M = Li	-10.43	10.43	6.81×10^3	1.73×10^4	-1.55×10^4
M = Na	-9.97	9.97	6.04×10^3	1.52×10^4	-1.39×10^4
M = K	-20.39	20.39	3.84×10^4	1.00×10^5	-8.24×10^4
($Ca@C_{50}N_5H_5$)-M	(M' = Ca)				
M = Li	-8.39	8.39	1.45×10^4	3.73×10^4	-3.18×10^4
M = Na	-7.88	7.88	1.18×10^4	2.98×10^4	-2.71×10^4
M = K	-11.63	11.63	1.09×10^5	2.66×10^5	-2.62×10^5

Table S2. The main components of the polarizability (α, au) and hyperpolarizability (β, au) tensor.

	Average polarizability				Magnitude of first hyperpolarizability			
	α_{xx}	α_{yy}	α_{zz}	α_0	β_{xxx}	β_{yyy}	β_{zzz}	β_0
$(Be@C_{50}N_5H_5)$-Li								
λ = ∝ (nm)	537	537	730	601	-5	-2	-54287	58423
λ = 1907	544	544	822	637	-6	-3	-68064	73095
λ = 1460	551	551	1443	848	-13	-7	-901505	915029
λ = 1340	555	555	196	435	-18	-11	230804	212737
λ = 1064	563	563	720	615	226	73	396426	586862
$(Be@C_{50}N_5H_5)$-Na								
λ = ∝	730	548	548	608	9	2	-33321	40164
λ = 1907	781	556	556	631	14	23	-9550	13581
λ = 1460	894	562	562	673	55	12	-52008	72224
λ = 1340	1630	565	565	920	26	34	-1175050	1188543
λ = 1064	749	579	579	636	149	175	-384784	474740
$(Be@C_{50}N_5H_5)$-K								
λ = ∝	825	581	581	662	26	72	-45538	65629
λ = 1907	889	595	595	693	44	135	-121303	173734
λ = 1460	950	607	607	721	175	397	-237856	317259
λ = 1340	985	613	613	737	613	939	-721747	936711
λ = 1064	1187	643	643	824	-63	-338	261658	339138
$(Mg@C_{50}N_5H_5)$-Li								
λ = ∝	542	542	857	647	-2	-3	-63490	66487
λ = 1907	550	550	1667	922	-5	-6	2371657	2371657
λ = 1460	556	556	578	563	-7	-8	63026	63026
λ = 1340	559	559	690	603	-37	-35	-887154	919949
λ = 1064	560	560	924	681	5	10	36734	43077
$(Mg@C_{50}N_5H_5)$-Na								
λ = ∝	869	558	558	662	1	3	-57964	66540
λ = 1907	-4502	567	567	-1123	15	108	-62746000	62794071
λ = 1460	657	574	574	601	4	11	-17544	2883
λ = 1340	725	578	577	627	6	-4	-124717	81690
λ = 1064	894	584	584	687	-388	-1950	9086540	9331842
$(Mg@C_{50}N_5H_5)$-K								
λ = ∝	1238	590	612	813	735	-17	-192692	219830
λ = 1907	918	610	640	723	690	143	179231	225621
λ = 1460	915	577	692	728	5826	-282	77871	153410
λ = 1340	1047	609	812	823	5344	886	549956	759743
λ = 1064	1086	630	912	876	-380	3349	-430626	577523
$(Ca@C_{50}N_5H_5)$-Li								
λ = ∝	548	574	1908	1010	39	1945	-682118	709910
λ = 1907	552	586	913	684	48	399	1983240	1993534
λ = 1460	568	611	1097	759	5	389	74239	73703
λ = 1340	685	647	1341	891	-54	-22093	-65582	110632
λ = 1064	572	520	1484	859	-201	-305	-235716	266417
$(Ca@C_{50}N_5H_5)$-Na								
λ = ∝	966	554	618	713	239	-1539	-24424	145624
λ = 1907	1166	579	601	782	15	15	150652	152250
λ = 1460	932	587	619	713	93	2	-69774	78760
λ = 1340	1163	592	635	797	436	251	-117834	121796
λ = 1064	2805	586	539	1310	-1876	-174	1154340	1100815
$(Ca@C_{50}N_5H_5)$-K								

$\lambda = \propto$	1845	592	624	1020	-853	761	-382650	405024
λ = 1907	871	657	659	729	1947	-18886	-5099470	4692958
λ = 1460	1017	586	755	786	141	-1092	-286002	386422
λ = 1340	1442	599	-781	420	150887	-6351	-38737	760043
λ = 1064	1400	628	597	875	560	-273	410793	540068

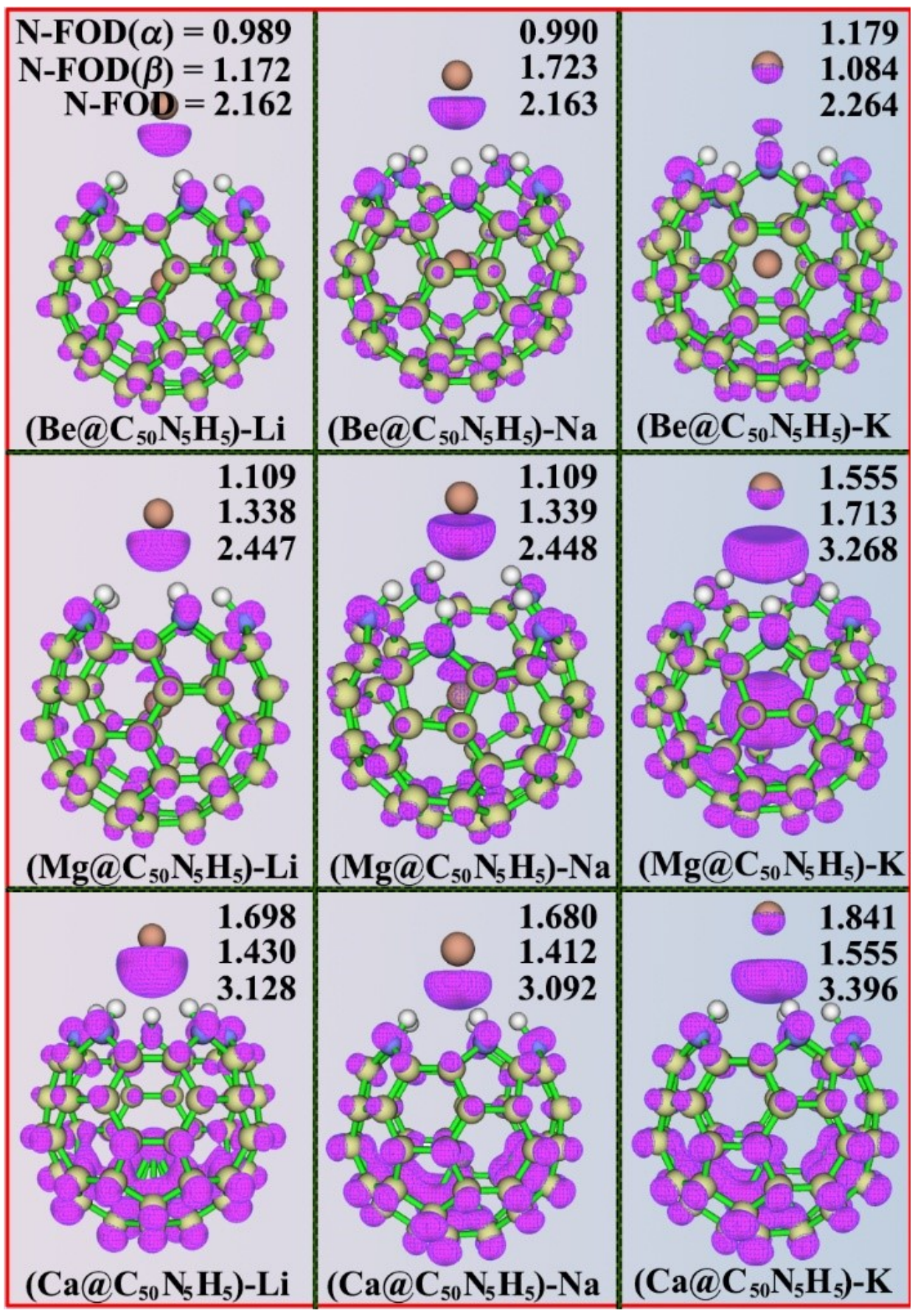

Fig. S1. Fractional orbital density (FOD) plot at σ = 0.003 e/Bohr^3 (TPSS/def2-TZVP (T =5000 K) level) for (M'@$C_{50}N_5H_5$)-M (M = Li, Na & K; M' = Be, Mg & Ca) (FOD depicted in pink).
